# Iron overload induces cerebral endothelial senescence in aged mice and in primary culture in a sex‐dependent manner

**DOI:** 10.1111/acel.13977

**Published:** 2023-09-07

**Authors:** Brian Noh, Maria Pilar Blasco‐Conesa, Syed Mushfiqur Rahman, Sheelu Monga, Rodney Ritzel, Gary Guzman, Yun‐Ju Lai, Bhanu Priya Ganesh, Akihiko Urayama, Louise D. McCullough, Jose Felix Moruno‐Manchon

**Affiliations:** ^1^ Department of Neurology McGovern Medical School at the University of Texas Health Science Center at Houston Houston Texas USA; ^2^ Solomont School of Nursing Zuckerberg College of Health Sciences University of Massachusetts Lowell Lowell Massachusetts USA

**Keywords:** cellular senescence, cerebral endothelial cells, iron overload, molecular biology of aging, sex characteristics

## Abstract

Iron imbalance in the brain negatively affects brain function. With aging, iron levels increase in the brain and contribute to brain damage and neurological disorders. Changes in the cerebral vasculature with aging may enhance iron entry into the brain parenchyma, leading to iron overload and its deleterious consequences. Endothelial senescence has emerged as an important contributor to age‐related changes in the cerebral vasculature. Evidence indicates that iron overload may induce senescence in cultured cell lines. Importantly, cells derived from female human and mice generally show enhanced senescence‐associated phenotype, compared with males. Thus, we hypothesize that cerebral endothelial cells (CEC) derived from aged female mice are more susceptible to iron‐induced senescence, compared with CEC from aged males. We found that aged female mice, but not males, showed cognitive deficits when chronically treated with ferric citrate (FC), and their brains and the brain vasculature showed senescence‐associated phenotype. We also found that primary culture of CEC derived from aged female mice, but not male‐derived CEC, exhibited senescence‐associated phenotype when treated with FC. We identified that the transmembrane receptor *Robo4* was downregulated in the brain vasculature and in cultured primary CEC derived from aged female mice, compared with those from male mice. We discovered that *Robo4* downregulation contributed to enhanced vulnerability to FC‐induced senescence. Thus, our study identifies *Robo4* downregulation as a driver of senescence induced by iron overload in primary culture of CEC and a potential risk factor of brain vasculature impairment and brain dysfunction.

## Graphical abstract

The brain function of aged female mice is more vulnerable to chronic administration of iron supplement ferric citrate (FC), compared with aged male mice. Senescence‐associated phenotype is exacerbated by FC in cultured primary cerebral endothelial cells (CEC) isolated from aged female mice, but not in CEC from aged male mice. Robo4 is downregulated in the cerebrovasculature of aged female mice, versus males, and CEC from aged male mice become more susceptible to FC after Robo4 downregulation.

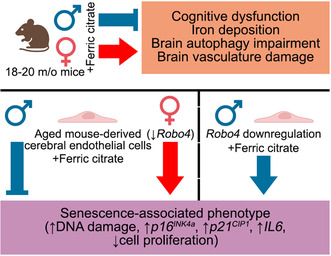

**Abbreviations**
BBB: blood brain barrier
CEC: cerebral endothelial cells
FC: ferric citrate
m/o: months old
SA: senescence associated

## 1 INTRODUCTION

The proper balance of iron uptake and release is critical for brain function (Hare et al., 2013). On one hand, iron deficiency negatively affects both the brain connectome (Algarin et al., 2017; Li et al., 2021) and hippocampal plasticity (Nelissen et al., 2017). Iron deficiency is associated with fibromyalgia (Yao et al., 2021) and anxiety (Li et al., 2011). On the other hand, with aging, iron levels increase in the brain (Sato et al., 2022), which contributes to oxidative stress, cell death, and brain damage (Yan et al., 2021). While iron deficiency has been extensively studied, there has been less investigation of the effects of iron overload. Importantly, elevated brain iron may contribute to Alzheimer's and Parkinson's diseases, and other neurological disorders (Ndayisaba et al., 2019).

Cerebral endothelial cells (CEC) are the major component of the most restrictive barrier between the circulating blood and the brain. Thus, any deleterious changes in CEC may initiate detrimental consequences for the entire brain (Marques et al., 2013). Cell senescence is a non‐proliferative state characterized by accumulation of DNA damage and the development of a pro‐inflammatory secretory phenotype that negatively affects the neighboring cells and tissues (Del Valle et al., 2009; Graves & Baker, 2020; Pelegrí et al., 2007; Yamazaki et al., 2016; Yang, Sun, et al., 2017; Yang, Yang, et al., 2017). Accumulation of senescent CEC occurs with aging (Kiss et al., 2020) and may alter the cerebrovasculature by dysregulating cerebral blood flow, increasing blood brain barrier (BBB) permeability, and changing the cerebrovasculature architecture (Del Valle et al., 2009; Yang, Sun, et al., 2017; Yang, Yang, et al., 2017).

Iron access to the brain occurs through the BBB and the blood cerebrospinal fluid barrier via transferrin‐mediated endocytosis. Iron overload and iron deposition are associated with cell senescence in cultured cell lines (Angelova & Brown, 2018; Killilea et al., 2003; Masaldan et al., 2018). However, whether iron overload induces senescence in primary culture of CEC and in the brain vasculature of aged mice have not been studied.

Ferric citrate (FC) is a common food additive and it is used as an oral iron supplement for the treatment of iron deficiency anemia in chronic kidney disease patients. FC treatment is very effective in increasing both hemoglobin and iron indexes in chronic kidney disease patients (Block et al., 2015; Fishbane et al., 2017; Lee et al., 2022). However, preclinical studies have found that FC can increase the risk of aluminum toxicity (Gupta, 2014) and induce neurodegeneration, and motor and cognitive deficits (Huang et al., 2019). As anemia is commonly found in the elderly population (≥65 years old) (Halawi et al., 2017), and this group has highest use of dietary supplements and prescription medications (Gahche et al., 2017), and unfortunately, as self‐medication is a frequent practice among the elderly population (Oliveira et al., 2018), the study of the effects of iron overload in aging models is clinically relevant.

In this study, we used aged (18–20 months old, m/o) mice of both sexes treated with FC (Gupta, 2014). We found that the brain function and the cerebrovasculature in aged female mice were more vulnerable to iron overload than in aged male mice. In in vitro experiments, we found that cultured primary CEC derived from aged female mice were more susceptible to become senescent by FC, compared with male CEC. RNA sequencing (RNA‐seq) revealed that the gene that codes for the endothelial transmembrane protein *Robo4* was downregulated in cultured CEC and in brain microvessel isolated from aged female mice, compared with those from male mice. Importantly, we found that *Robo4* downregulation sensitized male mouse‐derived CEC to become senescent by FC.

## 2 EXPERIMENTAL PROCEDURES

### 2.1 Animals and ethics statement and treatment

C57BL/6J male and female mice (18–20 m/o) from National Institute of Aging were housed in the animal facilities at the University of Texas McGovern Medical School. The experiments were conducted following the protocol approved by the Center for Laboratory Medicine and Care (protocol number AWC‐21‐0084) at the University of Texas McGovern Medical School. Mice were maintained in an environment with constant temperature and humidity on a 12 h light/12 h dark schedule, and with *ad libitum* access to water and mouse lab pellets. Researchers and veterinarians maintained mice with no distress, pain, or injury.

Mice were treated with 200 μL of 5% FC (in saline solution), or with 200 μL of physiological saline solution as control, by oral gavage alternatively 3 days per week for 6 weeks. Each cage contained FC‐ and vehicle‐treated mice. During the duration of the treatment, mice were maintained in the same conditions of temperature, humidity, light, and food/water as described above. All experiments were performed by investigators blinded to sex and treatment.

### 2.2 Reagents

Antibodies against actin (3700) were obtained from Cell Signaling. Antibodies against γH2AX (ab11174) were from Abcam. FC was from Sigma (F3388). Hoechst dye (#SC‐394039) and anti‐ICAM1 (#sc‐107) were from Santa Cruz Biotechnology. ON‐TARGETplus mouse Robo4 SMARTPool siRNA (L‐050278‐01) and ON‐TARGETplus mouse non‐targeting SMARTPool (D‐001810‐10) were from Horizon Discovery.

### 2.3 Isolation of cerebral endothelial cells from adult mouse brains and culture

Primary CEC were isolated from adult 18–20 m/o old C57BL/6J male and female mice using the Adult Brain Dissociation Kit (MACS Miltenyi Biotec, #130‐107‐677) as previously described (Noh et al., 2022). Briefly, brain tissue was mechanically and chemically homogenized, and filtered through 70 μm filters. Debris was separated by centrifugation, and red blood cells were removed. Then, the cell suspension was plated with Complete Mouse Endothelial Cell Medium/w Kit (Cell Biologics, #M1168) supplemented with puromycin (4 μg/mL) and maintained for 48 h to selectively maintain CEC in culture. After 48 h, medium was replaced with fresh complete medium. Complete medium contains 5% of fetal bovine serum, 0.1% VEGF, 0.1% ECGS, 0.1% heparin, 0.1% hydrocortisone, and 1% Antibiotic‐Antimycotic solution. For treatments, cultured CEC were treated with FC (50 or 150 μM) or a vehicle (H_2_O) in a final volume of 0.8 mL/cm^2^ for 7 days. Medium was not replaced during the treatments. For experiments with siRNA, cultured CEC were transfected with DharmaFECT (Horizon Discovery) and a total of 5 nM of siRNA against mouse Robo4 (Horizon), or non‐target siRNA as control.

### 2.4 Isolation of cerebral microvessels from adult mice

Isolation of microvessel fractions from adult mice was previously described (Noh et al., 2022). After obtaining the pellets with microvessels, the pellets were processed to isolate RNA and further perform qPCR or fixed and stained with antibodies.

### 2.5 Gene expression by qPCR

Cultured CEC or microvessel fractions were collected and processed following the manufacturer's instructions of the RNeasy Mini Kit (Qiagen, #74104) to isolate total RNA. RNA was reverse transcribed with the iScript Reverse Transcription SuperMix (Bio‐Rad, #1708840). cDNAs were then used for RNA‐Seq analyses (Qiagen) or used to analyze the gene expression by qPCR.

For qPCR analysis, cDNA was diluted in iTaq Universal SYBR® Green Supermix (Bio‐Rad, #1725121) and run using a Bio‐Rad CFX384 Touch device (95°C for 3 m, and 40 cycles of 95°C for 10 s, and 55°C for 30 s).

For RNA‐Seq analysis, log10 *p*‐value >2 was established as a variable to select the most significant differential expressed genes, and log2 fold change < −1.5 and >1.5 as a variable to select the most downregulated and upregulated differential expressed genes, respectively.

#### 2.5.1 Sequences of primers

Fw mouse Gapdh 5′‐CAAGGTCATCCATGACAACTTTG‐3′; Rv mouse Gapdh 5′‐GTCCACCACCCTGTTGCTGTAG‐3′. Fw mouse Robo4 5′‐GTCATTGCCAGTAGTGCTGTCC‐3′; Rv mouse Robo4 5′‐AATGGCGTCCTCGCTGGTGTAT‐3′. Fw mouse p16^INK4a^ (Cdkn2a) 5′‐TGTTGAGGCTAGAGAGGATCTTG‐3′; Rv mouse p16 5′‐CGAATCTGCACCGTAGTTGAGC‐3′. Fw mouse p21^CIP1^ (Cdkn1a) 5′‐TCGCTGTCTTGCACTCTGGTGT‐3′; Rv mouse p21 5′‐CCAATCTGCGCTTGGAGTGATAG‐3′. Fw mouse IL6 5′‐TACCACTTCACAAGTCGGAGGC‐3′; Rv mouse IL6 5′‐CTGCAAGTGCATCATCGTTGTTC‐3′. The relative expression of the gene of interest was calculated with the double delta Ct method related to the relative expression of *Gapdh*.

### 2.6 Immunocytochemistry

Cultured CEC and microvessels were fixed with 4% paraformaldehyde for 10 m at RT, and then permeabilized with 0.1% Triton‐X100 for 10 m at RT. Samples were blocked with 5% bovine serum albumin overnight at 4°C, and then incubated with a solution of primary antibodies in the blocking solution overnight at 4°C. Samples were incubated with Alexa Fluor®‐conjugated secondary antibodies (Abcam) for 1 h at room temperature, and with the Hoechst dye to stain nuclei.

Images from 5 to 10 microscopic fields (x20 or x40 objectives) were taken using the same exposure time and light intensity. We established threshold limits for each marker for quantitative analyses. The mean of fluorescence intensities of the GFP‐LC3‐RFP reporter and the standard deviation of γH2AX in each region of interest was quantified with ImageJ software. The fluorescence intensities from the background of each picture were subtracted from the correspondent values of the region of interest in the same image.

Images were taken with the EVOS FL 2 Auto Imaging System (Thermo Fisher Scientific).

### 2.7 Western blotting

Brain tissues from experimental mice were processed for electrophoresis and western blotting as previously described (Noh et al., 2022).

### 2.8 Immunohistochemistry

Brains from perfused and decapitated mice were fixed by immersion in 4% paraformaldehyde overnight at 4°C, and then dehydrated with 30% sucrose for 2 days at 4°C. Brains were sliced (24 μm thickness) with a freezing microtome, and sections were stored with an anti‐freezing solution at −20°C until needed. For Prussian blue staining, brain slices on glass slides were rehydrated with water and incubated with 20% hydrochloric acid–10% potassium ferrocyanide (1:1) solution for 20 m. After washing in distilled water, sections were counterstained with Nuclear fast red for 5 m. Then, slices were rinsed in distilled water and dehydrated with 95% and 100% ethanol and xylene. 20x magnification images were analyzed with Fiji‐ImageJ software to determine the number of iron deposits per area (mm^2^). Color channels were separated using the vector FastRed FastBlue DAB in the Colour deconvolution command. Then, the same threshold was applied for the blue channel of all images to identify the Prussian blue‐positive particles.

For γH2AX staining, brain slices were rehydrated with Tris‐buffered saline. Slices were incubated with 3% H_2_O_2_ for 30 m, blocked with 2% Donkey serum/PBS for another 30 m and then incubated with anti‐γH2AX (1:3000) overnight at 4°C. Then, samples were incubated with anti‐rabbit 1 h and developed with the 3,3′‐diaminobenzidine substrate kit (Vector laboratories, SK‐4100). Samples were washed and dehydrated with 95% and 100% ethanol and xylene. Color channels of 20x magnification images were separated using the vector H DAB in the Colour deconvolution command. Then, the same threshold was applied for the brown channel of all images to identify the γH2AX‐positive particles.

All images from immunohistochemistry studies were obtained on a Keyence BZ‐X810 microscope.

### 2.9 Measurement of iron content

Brain tissues and blood cell pellets were homogenized with 800 μL of 1 mmol/L HCl–10% trichloroacetic acid solution, and incubated for 1 h at 50°C with shaking. Lysates were centrifuged at 15,000 g for 15 m at RT, and the supernatants were collected. Supernatant (90 μL) was mixed with 30 μL of ascorbic acid (20 mg/mL), and then added 20 μL of a ferrozine solution (0.85% w/v ferrozine in hydroxylamine hydrochloride). Samples were incubated for 30 m at RT and the absorbance was measure at 560 nm. We used Iron Standard (Sigma, #02583) for the standard curve.

### 2.10 Flow cytometry and ex vivo SA‐β‐gal‐activity assay

Mice (18–20 m/o) were perfused with cold PBS, and the left hemisphere was isolated for single cell processing as described in (Ritzel et al., 2022). Briefly, brains were placed in RPMI 1640 (Invitrogen, #22400105) medium and mechanically and enzymatically digested for 45 m at 37°C on a shaking incubator. The cell suspension was filtered through a 70‐μm filter, and cells were then incubated with TruStain FcX Block (Biolegend, #101320), for 10 m on ice, and stained with CD45‐eF450 (eBioscience, #48–0451‐82), CD31‐PECy7 (Biolegend, #102444), and Tie2‐APC (Biolegend, #124010). The fixable viability dye Zombie Aqua was used for live/dead discrimination (Biolegend, #423102). Cells were then washed in FACS buffer, fixed in 2% paraformaldehyde for 8 m, and washed once more prior to adding 500 μL FACS buffer. Senescence associated (SA) β‐Galactosidase Activity Assay Kit was performed according to the manufacturer's instructions (Cell Signaling Technologies, #35302S).

Data were acquired on a Beckman Coulter CytoFLEX LX cytometer using CytExpert v2.5 (Beckman Coulter) and analyzed using FlowJo (Treestar Inc.). Cells were first gated using a splenocyte reference (SSC‐A vs. FSC‐A). Singlets were gated (FSC‐H vs. FSC‐W), and live cells were gated based on Zombie Aqua exclusion (SSC‐A vs. Zombie Aqua‐Bv510). Brain endothelial cells were identified as the CD45^−^CD31^+^Tie2^+^ population. A tissue‐ and cell type‐matched fluorescence minus one control was used to determine gating for SA‐β‐Gal‐activity.

### 2.11 Scratch assay

Cultured CEC 100% confluent were scratched with a 200 μL tip. Cells were washed with PBS, and medium with the corresponding treatments was added. The plate was placed in the Incucyte Live‐Cell Analysis System (Sartorius), and cells were imaged every 8 h for 48 h. Distance between edges was measured at five different intervals per image using the ImageJ software. The percentage of the wound closure per time point was calculated relative to the first image taken for each well.

### 2.12 Behavior

Y‐maze: a mouse was placed in the interjection of the three arms of a Y‐shaped structure (39.5 × 8.5 × 13 cm), and allowed it to move freely through the maze during a 5 m session. Movements were video recorded, and a blinded investigator to the experimental groups analyzed the number of arm entries. The percentage of spontaneous alternation was calculated as [(number of alternations)/(total arm entries − 2)] × 100.

Open Field: a mouse was placed in a squared arena (40 cm per side) and allowed it to explore for 20 m. Movements were video recorded and velocity, distance moved, time spent in the center and borders of the arena, and the frequency of visits to these areas were automatically calculated by the Ethovision XT software.

Novel object recognition: 24 h after the Open Field test, two identical objects were presented to a mouse in the arena, and the mouse was allowed to explore the objects for 10 m. Twenty‐four hours later, one of these objects was replaced with a novel object, and again, the mouse was allowed to explore the novel and the familiar objects for 10 m. We evaluated the differences in the exploration time with novel and familiar objects by calculating the recognition index as [(time with novel object)/(time with novel object + time with familiar object)].

Fear conditioning test: A mouse was placed in an arena (novel environment, 30 cm × 20 cm) in which does not enter light from the room, only a white light installed inside the arena (neutral stimulus). The mouse was allowed to explore for 2 m. 1 h later, the animal was again placed in the arena with the same light and allowed to explore other 2 m, time that was followed by a 1 mA electric foot shock (aversive stimulus) for 2 s. Twenty‐four hours after the aversive stimulus, the animal was returned to the arena and exposed to the light without shock. The percentage of inactive time was recorded.

### 2.13 Statistics

All analyses were performed using GraphPad Prism software (v.7). We used Student's *t* test to compare means from two independent groups, and two‐way ANOVA to compare groups with two variables, such as sex and treatment (Table S1). We used Tukey's test for multiple comparisons. A value of *p* < 0.05 was considered significantly different. Bar graphs represent mean ± SEM.

## 3 RESULTS

### 3.1 Cognitive function was more vulnerable to iron overload in aged female mice than in aged male mice

**FIGURE 1:**
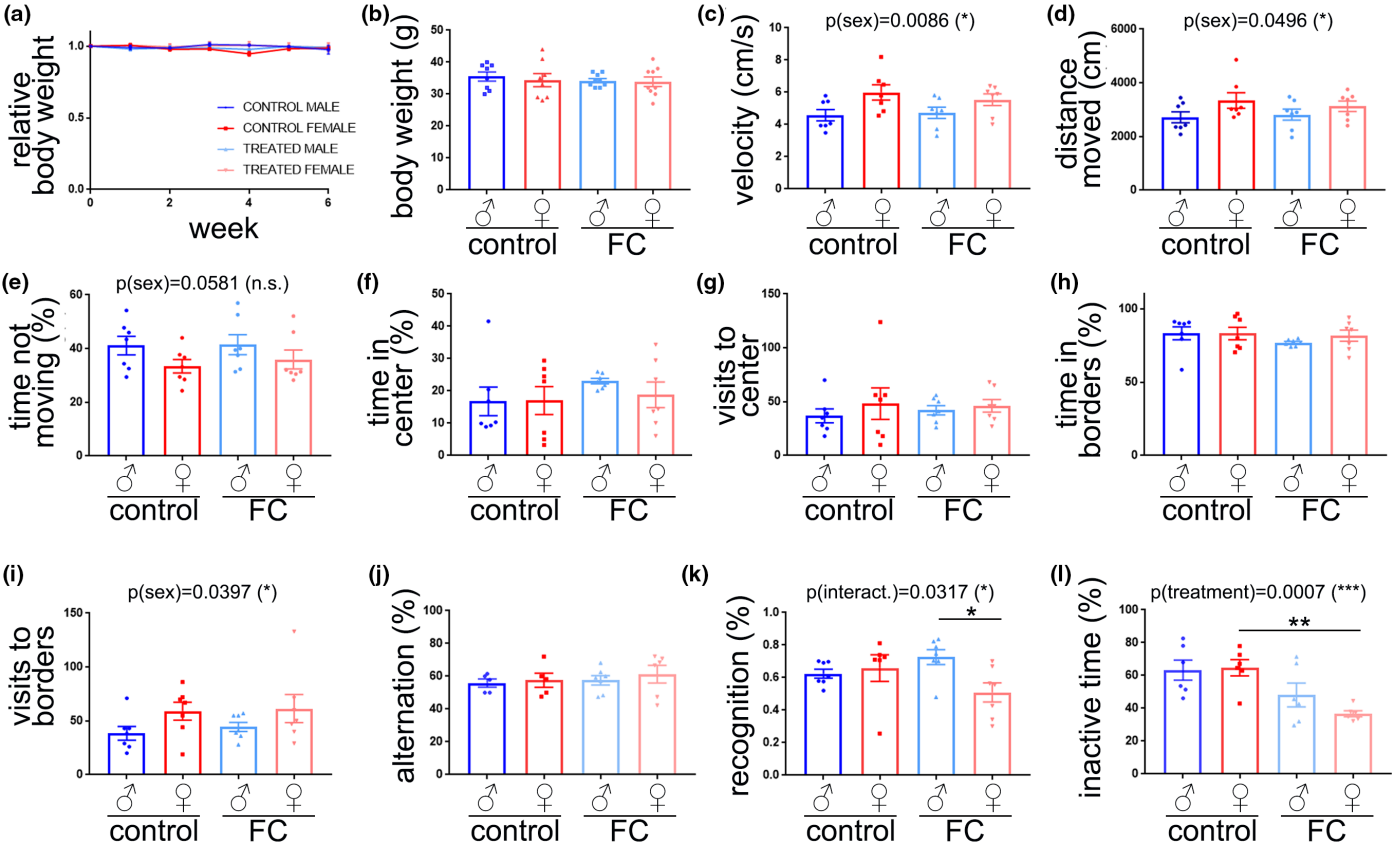
Iron overload impaired recognition and conditioned memory in aged female mice. (a) Relative body weight in mice from FC‐treated and control groups. (b) Absolute body weight 6 weeks after the initiation of the treatments. (*c–i*) Open field: velocity (c), distance moved (d), percentage of time not moving (e), percentage of time spent in the center (f), number of visits to the center (g), percentage of time spent in the borders (h), and number of visits to the borders (i). (j) Percentage of alternation. (k) Novel object recognition index. (l) Percentage of freezing time. All data are mean ± SEM with *n* = 6–8. Two‐way ANOVA test, Tukey's multiple comparisons test, *p < 0.05, **p < 0.01.

We measured body weight in 18–20 m/o mice of both sexes treated with FC, and found no significant differences between groups (Figure 1a,b). We tested mice for motor skills and anxiety‐like behavior by Open Field test, and found that treatment did not have significant effect. However, sex had a significant effect on velocity (Figure 1c) and distance moved (Figure 1d), being female mice what showed higher velocity and moved longer distances, compared with males. Aged female mice spent less time not moving than aged male (Figure 1e). Furthermore, we did not find differences between sexes or treatment in time spent either in the center of the arena (Figure 1f) or in borders (Figure 1h). Importantly, sex had a significant effect on the number of visits to borders, being female mice what visited this area more often than males (Figure 1i). Post hoc test did not detect significant differences between the four groups in the variables mentioned above.

We tested spatial memory, recognition memory, and conditioning memory with the Y‐maze, novel object recognition, and fear conditioning tests, respectively. There were no differences in percentage of alternation (Figure 1j). However, we found that the recognition memory was significantly affected by an interaction between treatment and sex (Figure 1k), and that female mice treated with FC showed a significant reduction in the recognition index percentage, compared with FC‐treated males. We did not find significant differences in exploration time with familiar and novel objects between sexes (Figure S1). The conditioning memory was significantly reduced by the treatment with FC (Figure 1l), and FC‐treated female mice showed reduced freezing time compared with control females. Interestingly, there were no significant changes in freezing time between treated and control males. Our data indicate that recognition memory and conditioning memory are more importantly altered by chronic administration of FC in aged female mice, compared with aged males.

**FIGURE 2:**
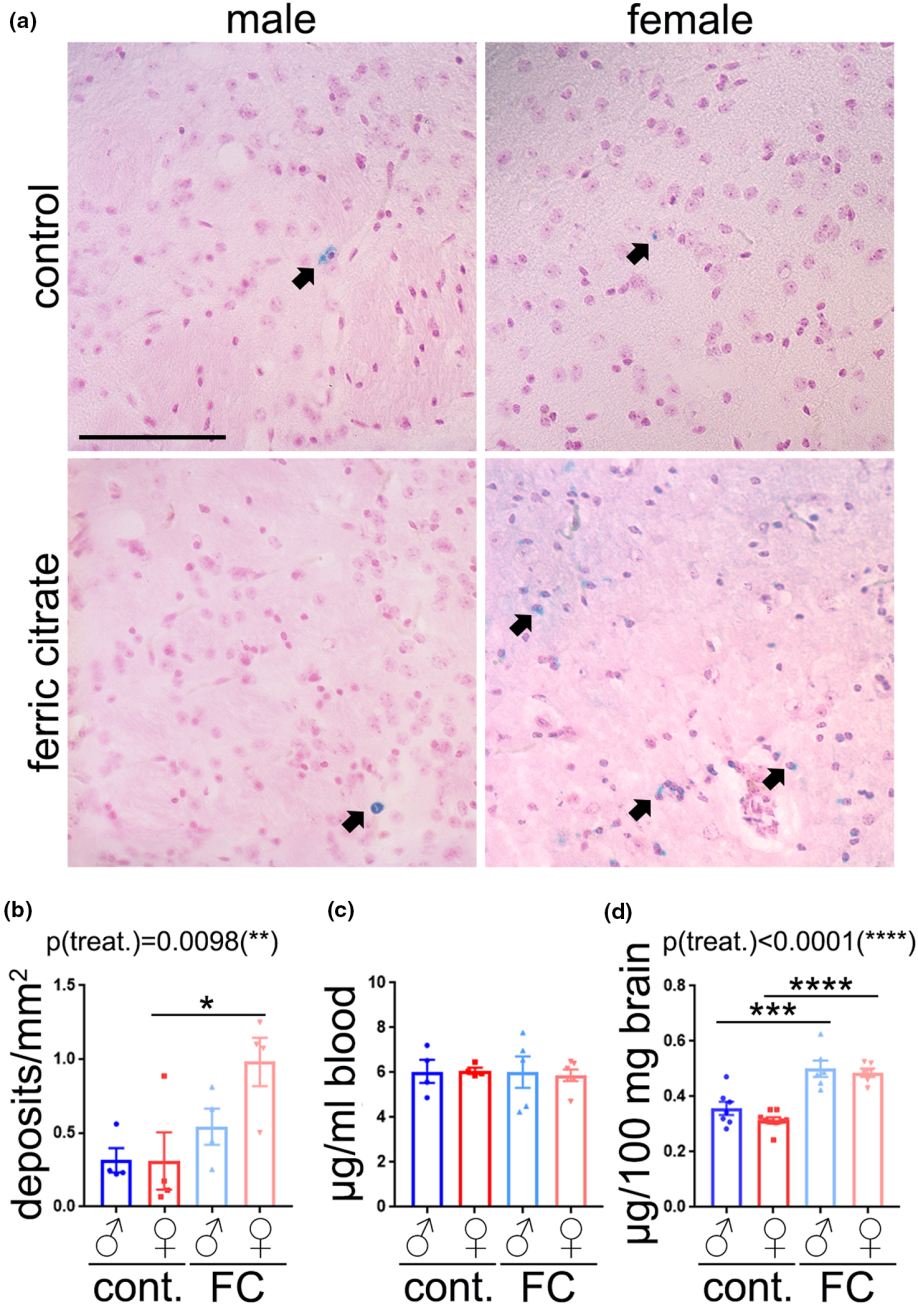
FC treatment enhanced iron deposition in brains of aged female mice, but not in male mouse brains. (a) Representative images of brain sections (cortex region) from aged male and female mice treated with FC or a vehicle stained with Prussian blue (blue) and Nuclear Fast Red (pink). Black arrows depict discrete iron deposits. Scale bar, 100 μm. (b) Number of Prussian blue‐positive deposits per area from (a). Six brain sections per mouse were stained and quantified. Data are mean ± SEM with *n* = 4. Two‐way ANOVA test, Tukey's multiple comparisons test, *p < 0.05. (c) Iron content in blood cells of mice of the experimental groups. (d) Iron content in the brain lysates of the experimental groups. Data are mean ± SEM with *n* = 7–8. ***p < 0.001, ****p < 0.0001. Data are mean ± SEM with *n* = 4–6.

As iron can accumulate in the form of iron deposits in the brain with aging, and iron deposition has been associated with brain dysfunction (del C Valdés Hernández et al., 2015), we determined whether iron overdose contributes to iron deposition in the brain in a sex‐dependent manner. Brain samples were stained with Prussian blue to visualize iron deposits. These iron deposits were found in the hippocampus, thalamus, cortex, and striatum (Figure S2). FC treatment had a significant effect on brain iron deposition (Figure 2a,b). The number of iron deposits increased in the brains of female mice treated with FC, compared with control females. However, no significant changes were observed between groups of aged male mice. Iron content in blood cells was not different between groups (Figure 2c); however, FC treatment enhanced the levels of iron in the brain of aged mice in both sexes (Figure 2d).

### 3.2 Iron overload negatively affected the brain vasculature of aged female mice

Given that iron overload can induce senescence in different cell types in culture (Angelova & Brown, 2018; Cozzi et al., 2019; Curtis et al., 2001; Yang, Sun, et al., 2017; Yang, Yang, et al., 2017; Yuan et al., 2021), and that senescence in the brain has been associated with cognitive deficits (Graves & Baker, 2020; Lin et al., 2021; Sikora et al., 2021), we hypothesized that FC‐induced cognitive dysfunction in females is caused by enhanced senescence in the brain.

Autophagy impairment is one of the features associated with cell senescence (Cayo et al., 2021; Rajendran et al., 2019; Xu et al., 2020). Thus, we analyzed the levels of several autophagy markers in mouse brain lysates (Figure S3a). Treatment had a significant effect on Beclin1 (Figure S3b), Atg7 (Figure S3c), p62 (Figure S3e), Lamp1 (Figure S3f), and LC3‐II (Figure S3d). p62 was significantly affected by sex. FC treatment increased p62 levels in both sexes, and only female mice treated with FC showed significant increases in Lamp1 compared with control female, but no changes were observed in male mice with FC treatment. Altogether, these data suggest that FC stimulates the initial stages of autophagy, but it negatively interferes in the later stages of autophagy, an effect that was more notable in aged female mice than in males.

**FIGURE 3:**
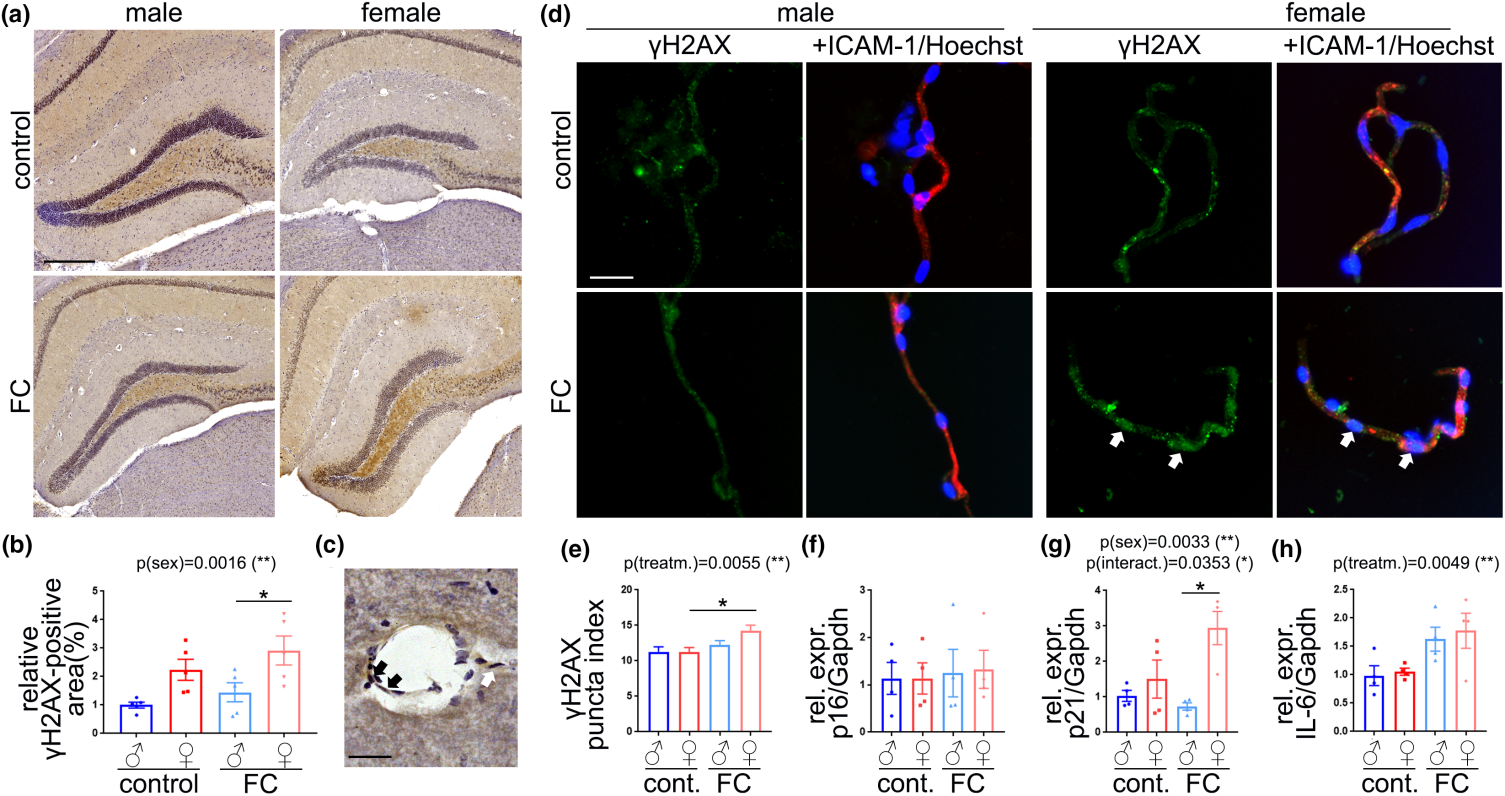
The brain vasculature of aged female mice was more vulnerable to FC than that of aged male mice. (a) Representative images of the hippocampi of mice from control or FC groups stained with anti‐γH2AX (brown) and Hematoxylin (purple). Scale bar, 250 μm. (b) Relative area positive to γH2AX from (a). Data are mean ± SEM with *n* = 5. (c) Representative image of a cerebral vessel positive to γH2AX (black arrows). Scale bar, 25 μM. (d) Representative images of cerebral microvessels from mice from control or FC groups stained with anti‐γH2AX (green) anti‐ICAM‐1(red) and Hoechst (blue). Scale bar, 25 μM. (e) Puncta index of γH2AX from (d). Data are mean ± SEM pooled from >100 microvessels from four mice/group. (f–h) Gene expression of *p16* (f), *p21* (g), and *IL6* (h) relative to *Gapdh*. Data are mean ± SEM pooled from four mice/group. All experiments were analyzed by two‐way ANOVA test, Tukey's multiple comparisons test, *p < 0.05.

DNA damage is the most important contributor to cell senescence (Yousefzadeh et al., 2021). Thus, we determined if chronic administration of FC induces DNA damage in the brains of aged mice of both sexes. Brain samples were stained with antibodies against γH2AX, commonly used as a marker of double strand DNA damage, the most deleterious form of DNA damage (He et al., 2016) (Figure 3a). We analyzed the hippocampus as recognition and conditioning memories are regulated by this brain area (Broadbent et al., 2004; Kim & Cho, 2020). The percentage of brain area positive to γH2AX was more pronounced in female mice than in male in control condition, and, importantly, FC significantly exacerbated differences between sexes (Figure 3b).

Further examination of the brain images revealed that the blood vessels can be positive to γH2AX staining (Figure 3c, arrows). To further investigate the effects of FC particularly on cerebral microvessels, we isolated the microvessel fractions from the mouse brains. We measured the puncta index of γH2AX, which represents the distribution of γH2AX as discrete puncta (DNA lesions) when the index is higher (Moruno‐Manchon et al., 2017). The puncta index of γH2AX was significantly increased in the microvessel fraction of aged female mice treated with FC, compared with control female, whereas we did not find significant differences between groups in the microvessels from male mice (Figure 3d,e). We also analyzed the relative gene expression of p16^Ink4a^ (*Cdkn2a*), p21^Cip1^ (*Cdkn1a*), and IL6 from the microvessel fractions derived from female and males treated with FC or a vehicle (Figure 3f–h). Sex had a significant effect on p21^Cip1^ expression (Figure 3g), and treatment had a significant effect on IL6 (Figure 3h). We also found an interaction between treatment and sex in p21^Cip1^ expression as well (Figure 3g). Microvessel fractions of aged female mice treated with FC showed enhanced expression of p21^Cip1^, compared to the microvessel fractions from treated male mice.

Given that endothelial senescence can negatively affect brain vasculature integrity, we wondered if FC induces vasculature impairment. We analyzed the levels of two common tight junction proteins: ZO‐1 and claudin‐5 (Figure S4). We did not find significant differences in ZO‐1 levels between groups (Figure S4b). However, sex had a significant effect on the levels of claudin‐5 **(**Figure S4c), and claudin‐5 was significantly increased in the brains of female mice treated with FC, compared with treated male mice.

**FIGURE 4:**
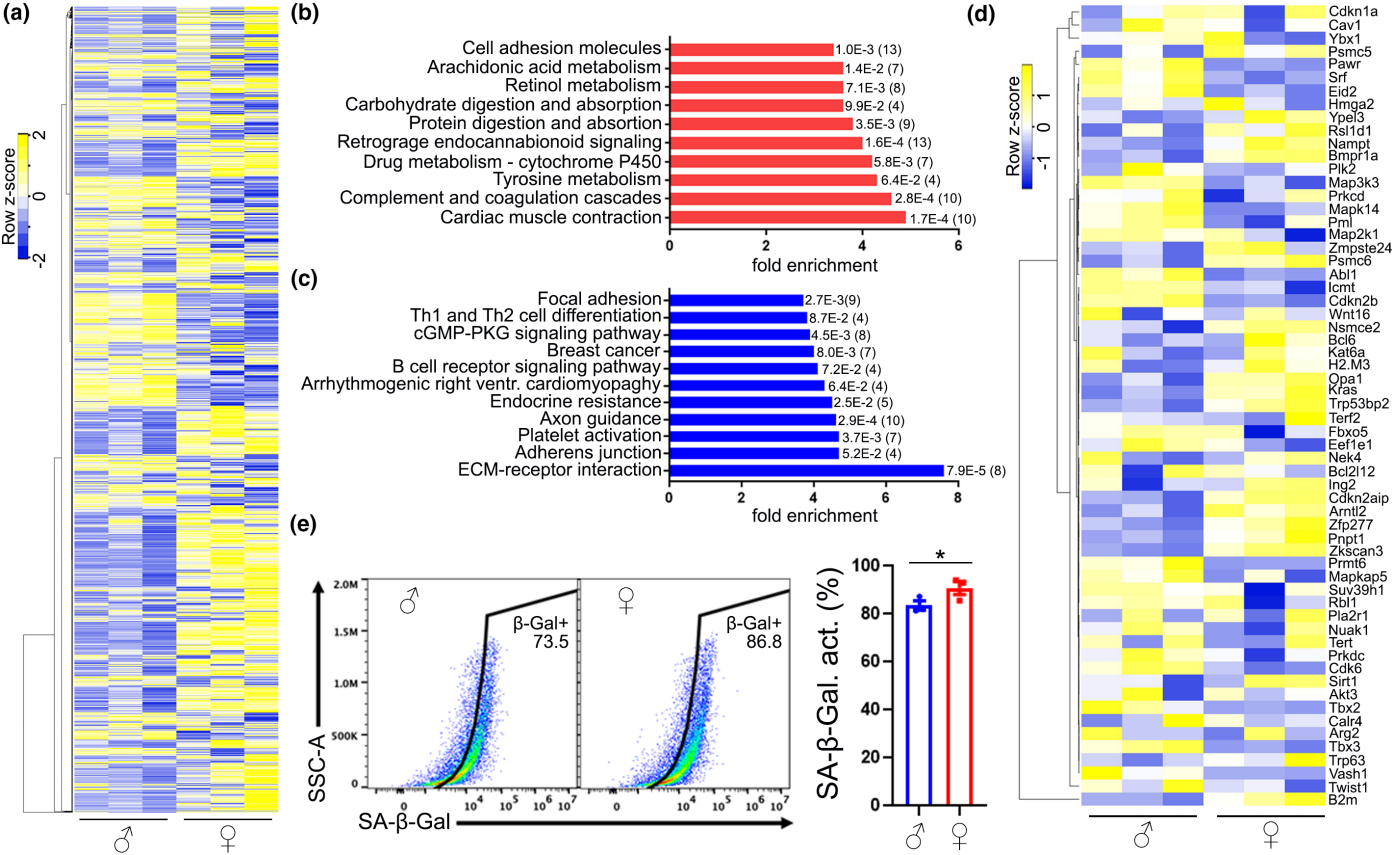
The transcriptome of cultured CEC isolated from aged mice showed sex differences. (a) Heatmap of the 2500 most expressed genes in cultured CEC isolated from 18 to 20 m/o male and female mice. (b,c) Representation of the most enriched KEGG pathways containing the most significant upregulated (b) and downregulated (c) genes in aged female‐derived CEC, compared with aged male‐derived CEC. Each KEGG pathway shows a p‐value and, in brackets, the number of genes in each group. (d) Heatmap of a SA genes in cultured CEC isolated from 18 to 20 m/o male and female mice. (e) Dot plot and bar graph of the percentage of SA‐β‐Galactosidase activity in CD31‐positive cells isolated from aged male and female mice. Data are mean ± SEM pooled from three mice/group. Student's *t* test, *p < 0.05.

This evidence suggests that the brain vasculature of aged female mice is more vulnerable to FC than that of aged males.

### 3.3 Cultured primary CEC derived from aged mice showed sex differences in SA phenotype

We then sought to identify the molecular mechanism/s that induce sex‐biased senescence in the brain vasculature of aged mice treated with FC. We used an in vitro model of cultured CEC derived from 18 to 20 m/o mice. We previously showed that our primary culture of CEC conserves important characteristics of the cerebrovasculature in mice, and that it is relevant to study mechanisms related to aging (Noh et al., 2022).

First, we analyzed the transcriptome profiles of these cells by RNA‐Seq. CEC isolated from aged male and female mice showed robust differences (Figure 4a). We identified 1202 and 315 differential expressed genes upregulated and downregulated, respectively, in CEC derived from aged female mice, compared with CEC from aged male mice. We found that genes involved in cardiac muscle contraction, complement and coagulation cascades, and in metabolism were the most significant upregulated genes in aged female mouse‐derived CEC, compared with aged male CEC (Figure 4b). Genes involved in extracellular matrix‐receptor interactions, adherens junction and focal adhesion, and platelet activation were significantly downregulated in aged female‐derived CEC compared with male CEC (Figure 4c).

Next, we aimed to determine if there are sex differences in SA phenotype. In a heat map of genes associated with cell senescence (Figure 4d), we found that female‐derived CEC, compared with male‐derived CEC, showed upregulated expression of multiple genes that promote senescence, such as *Nampt* (Ma et al., 2017), *Opa1* (Tezze et al., 2017), *KRas* (Lee & Bar‐Sagi, 2010), *Zfp277* (Negishi et al., 2010), and *Zkscan3* (Hu et al., 2020). In addition, female CEC also showed downregulated expression of genes associated with senescence prevention, such as *Srf* (Ding et al., 2001), *Map3k3* (Zhou et al., 2022), *Abl* (Zhang et al., 2013), *Prmt6* (Stein et al., 2012), *Cdk6* (Ruas et al., 2007), and *Tbx3* (Kumar et al., 2014). Furthermore, we found by flow cytometry that a higher percentage of cells positive to the endothelial marker CD31 isolated from aged female mice were positive to SA‐β‐Galactosidase, compared with CD31‐positive cells from aged male mice (Figure 4e). This supports our hypothesis that endothelial cells derived from aged female mice are more susceptible to enter into a senescent state, compared with endothelial cells derived from aged male mice.

### 3.4 Cultured primary CEC derived from aged female mice were more vulnerable to FC than CEC from aged male mice

**FIGURE 5:**
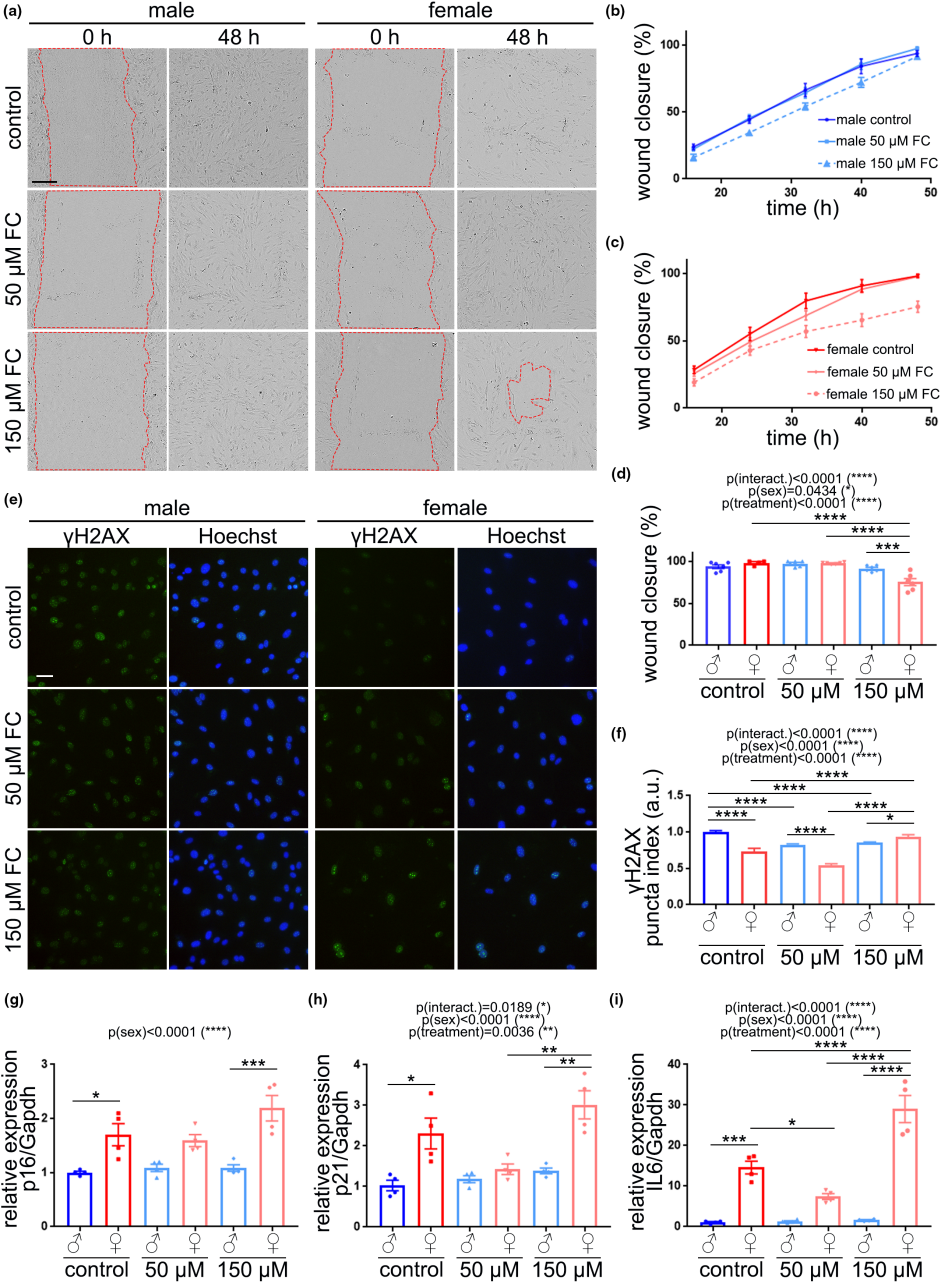
Cultured primary CEC isolated from aged female mice were more susceptible to FC than CEC from male mice. (a) Representative images of the scratch assay in cultured CEC isolated from aged male and female mice and treated with FC (50 or 150 μM), or a vehicle. Images show wound right after (0 h) and 48 h after (48 h) a scratch and treatment initiation. Scale bar, 200 μm. (b,c) Percentage of the wound closure in male CEC (b) and female CEC (c) treated with FC or a vehicle. (d) Percentage of wound closure 48 h after the initiation of the treatments. Data are mean ± SEM pooled from three mice/group. (e) Representative images of cultured CEC from male and female mice treated with FC (50 or 150 μM), or a vehicle. 7 days after the treatments, cells were fixed and stained with anti‐γH2AX (green) and the nuclear Hoechst dye (blue). Scale bar, 25 μm. (f) Puncta index of γH2AX in CEC from (e). Data are mean ± SEM pooled from >500 cells from three mice/group. (g–i) Gene expression of *p16* (g), *p21* (h), and *IL6* (i) relative to *Gapdh*. Data are mean ± SEM pooled from three mice/group. All experiments were analyzed by two‐way ANOVA test, Tukey's multiple comparisons test, *p < 0.05, **p < 0.01, ***p < 0.001, and ****p < 0.0001.

One of the characteristics of senescent endothelial cells is the inability to migrate properly (Liu et al., 2022; Yang et al., 2018). To analyze migration, we performed a scratch assay in CEC derived from aged male and female mice treated with FC (50, or 150 μM) or a vehicle (Figure 5a–c). Sex, treatment, and their interaction had significant effects on wound closure (Figure 5d), which was significantly reduced in aged female‐derived CEC treated with 150 μM FC, compared with male CEC at the same conditions.

Then, we wondered if FC may have an effect on cell proliferation, which is inhibited during cell senescence. To study cellular senescence in vitro, cells should be allowed to have at least 7 days after a senescence‐inducing stimulus to become fully senescent (Gonzalez‐Gualda et al., 2021). Thus, in cell proliferation/viability assays, we treated cells with FC (50, or 150 μM), or water as vehicle, for 24 h and 7 days. There were no significant differences 24 h after the treatment (Figure S5). However, 7 days after treatment, control female CEC showed a slight reduction in cell proliferation, compared with corresponding male CEC, and FC treatment significantly reduced cell proliferation in both sexes. Importantly, FC did not induce cell death in CEC derived from both sexes (Figure S6). We also determined if FC can alter autophagy in cultured CEC. For this, we used a construct that codes for an autophagy flux reporter (GFP‐LC3‐RFP) (Kaizuka et al., 2016; Morita et al., 2018). Both at 24 h and 7 d after treatment with FC or vehicle, autophagy flux was significantly reduced in male‐derived cells treated with 150 μM FC, compared with control male cells (Figure S7); however, female‐derived CEC did not exhibit any change in autophagy flux with FC treatment.

**FIGURE 6:**
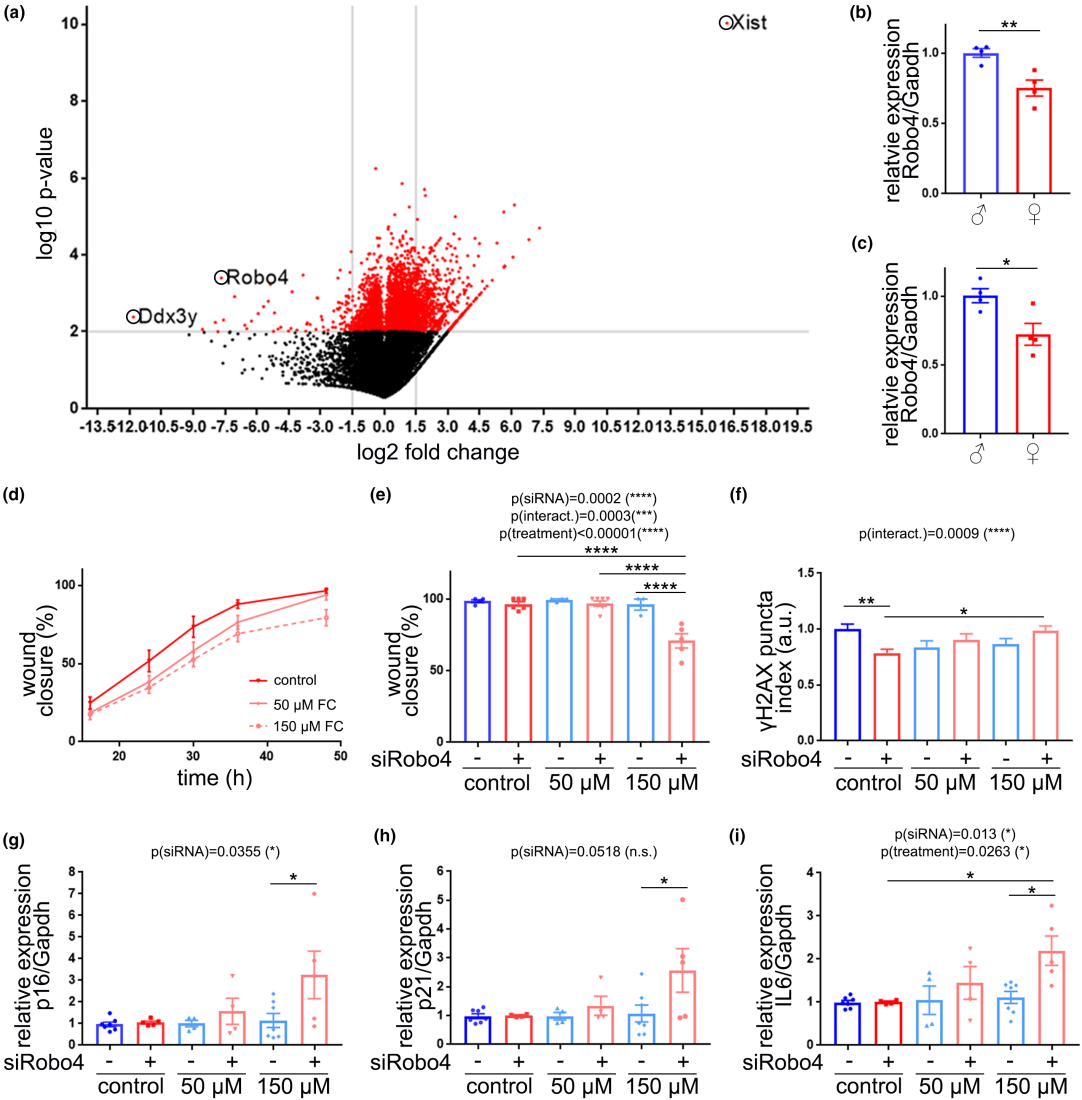
*Robo4* downregulation sensitized aged male mice‐derived CEC to FC. (a) Volcano plot showing fold changes for genes differentially expressed between aged female mouse‐derived CEC versus aged male mouse‐derived CEC in culture. (b) Gene expression of *Robo4* relative to *Gapdh* in cultured CEC from aged mice of both sexes. Data are mean ± SEM pooled from three mice/group. Student's *t* test, *p < 0.05. (c) Gene expression of *Robo4* relative to *Gapdh* in brain microvessel fractions from aged male and female mice. Data are mean ± SEM pooled from three mice/group. Student's *t* test, *p < 0.05. (d) Cultured CEC from male mice were transfected with siRNA targeting *Robo4*, or non‐targeting siRNA, and treated with FC, or a vehicle. Line graph shows the percentage of the wound closure every 8 h for 48 h. (e) Percentage of wound closure 48 h after the initiation of the treatments. (f) Puncta index of γH2AX in CEC 7 days after the treatments. Data are mean ± SEM pooled from >250 cells from three mice/group. Two‐way ANOVA test, Tukey's multiple comparisons test, *p < 0.05, **p < 0.01. (g–i) Gene expression of *p16* (g), *p21* (h), and *IL6* (i) relative to *Gapdh*. Data are mean ± SEM pooled from two independent experiments from three mice/group. Two‐way ANOVA test, Tukey's multiple comparisons test, *p < 0.05, **p < 0.01.

We determined if FC induces DNA damage in cultured CEC by measuring the puncta index of γH2AX, and observed that sex, treatment and interaction between both factors had significant effect on DNA damage (Figure 5e,f). Female CEC showed reduced γH2AX than male CEC at basal conditions; however, FC induced DNA damage in female CEC, but not in male CEC. Indeed, FC reduced γH2AX in male CEC, suggesting that FC has beneficial effects on male CEC and detrimental in female CEC.

Sex had a significant impact on the relative expression of p16^Ink4a^ (Figure 5g), p21^Cip1^ (Figure 5h), and IL6 (Figure 5i). The treatment and the interaction had significant effects on p21^Cip1^ and IL6. Remarkably, 150 μM FC increased the relative expression of p16^Ink4a^, p21^Cip1^, and IL6 in CEC derived from female mice compared with male CEC.

Altogether, our data indicate that FC exacerbates the SA phenotype in cultured female mouse‐derived CEC, and that CEC from aged male mice are more resilient to FC treatment.

### 3.5 Robo4 was downregulated in the cerebrovasculature and in cultured primary CEC derived from aged female mice compared with aged males

We use our RNA‐Seq data to identify candidate genes that could contribute to differences in senescence susceptibility between sexes (Moruno‐Manchon, 2023). In a volcano plot representation (Figure 6a), we observed that the most upregulated gene in aged female CEC is *Xist*, a non‐coding RNA on the X chromosome that participates in X‐inactivation. The most downregulated gene in aged female CEC is *Ddx3y*, which codes for an RNA helicase on the Y chromosome. Thus, these two genes confirm the different expression of sex‐linked genes and serve as positive controls for male‐ (*Ddx3y*) and female‐ (*Xist*) derived CEC. *Robo4* was the second most significantly downregulated gene in female‐derived CEC, compared with male CEC. We confirmed that *Robo4* was downregulated in cultured CEC isolated from aged female mice, compared with male CEC (Figure 6b). We also analyzed the relative expression of *Robo4* in the microvessel fractions of aged mice, and found that *Robo4* was downregulated in aged female mice, compared with aged males (Figure 6c).

### 3.6 Robo4 downregulation promoted FC‐induced senescence in primary cultured CEC from aged male

ROBO4 (Magic roundabout) is a transmembrane receptor protein that is specifically expressed in endothelial cells and plays a major function in cell proliferation and angiogenesis (Huminiecki et al., 2002). In preclinical studies, Robo4 has also been implicated in preventing senescence induced by TNFα (Tanaka et al., 2017). Thus, we hypothesize that *Robo4* downregulation may sensitize endothelial cells to become senescent. Then, we transfected male‐derived CEC with *Robo4*‐targeting siRNA, or non‐targeting siRNA (Figure S8). *Robo4* downregulation significantly reduced the percentage of the wound closure, compared with CEC treated with *Robo4*‐targeting siRNA, when treated with 150 μM FC (Figure 6d,e). *Robo4*‐targeting siRNA also negatively affected cell proliferation 24 h and 7 d after the initiation of FC treatment (Figure S8). Furthermore, *Robo4* downregulation significantly enhanced DNA damage in CEC treated with 150 μM FC, compared with siRobo4 CEC treated with a vehicle (Figure 6f). Regarding the expression of SA genes, we found that CEC treated with *Robo4*‐targeting siRNA and 150 μM FC showed significantly upregulated expression of p16^Ink4a^ (Figure 6g), p21^Cip1^ (Figure 6h), and IL6 (Figure 6i), compared with CEC treated with non‐targeting siRNA and 150 μM FC.

Thus, our data indicate that *Robo4* downregulation “sensitizes” male mouse‐derived CEC to enter into a senescent state, as we observed in female mouse‐derived CEC (Figure 5).

## 4 DISCUSSION

Our study demonstrates that the brains of aged female WT mice are more vulnerable to chronic administration of FC, compared to aged male mice. Aged female mice treated with FC manifest cognitive dysfunction, enhanced iron deposition, impaired autophagy, and enhanced SA‐phenotype in their brain vasculature. In our in vitro experiments, we found that cultured primary CEC isolated from aged female mice and treated with 150 μM FC are more susceptible to show SA phenotype, compared with CEC from aged male mice. Importantly, *Robo4* downregulation sensitizes male‐derived CEC to FC treatment to become senescent.

Different models have been used to study the toxicity of iron overload on brain functions. Iron solutions can be injected in the brain by intranigral infusion (Mohanakumar et al., 1994; You et al., 2015), or administered by oral supplementation (Huang et al., 2019; Schroder et al., 2001; Sobotka et al., 1996). Huang et al., used a chronic oral administration of FC (2.5 or 10 mg/day, >4 weeks) in 9 m/o C57BL/6 mice to study the toxicity of iron overload on brain function (Huang et al., 2019). As our bodies incorporate iron only from aliments, we found Huang's model more relevant; however, daily gavage in aged mice resulted in a high mortality independently of the treatment during the first week of treatment, likely because daily gavage is more stressful for aged mice than for young mice. Thus, we performed oral gavage of FC or saline solution alternatively 3 days per week. Huang et al. observed Parkinson‐like phenotype in middle aged male mice treated with FC for 4 and 8 weeks of treatment, compared with control mice. Six weeks after the initiation of the treatment, we observed significant differences in recognition and conditioning memory between FC‐treated female mice and control females, but not between both groups of males. Thus, we found relevant to investigate the molecular mechanisms that may drive to these different outcomes in a sex‐dependent manner, which have not been studied thus far.

Iron deposits have been found in the brain of healthy aged population, and more pronounced in age‐related neurodegenerative disorders (Ndayisaba et al., 2019). Importantly, iron accumulates in specific brain regions and correlates to cognitive dysfunction (Spence et al., 2020). It is still debatable whether iron is a primary cause of dementia or whether iron accumulation in the brain is a secondary effect of brain atrophy. Iron levels in blood are restored at basal levels 6 h after FC administration in rats (Yuan et al., 2017). However, we found that iron levels are enhanced in the brains after chronic administration of FC, suggesting that iron accumulates in the brain tissue during the treatment. Senescence itself can promote iron accumulation (Killilea et al., 2003; Killilea et al., 2004). Thus, aged brains, which show SA phenotype, compared with the brains of young mice (Kiss et al., 2020), may contribute to iron accumulation. In endothelial cells, the transmembrane protein neuropilin‐1 prevents iron accumulation in mitochondria and, thus, mitigates iron‐induced oxidative stress and cell senescence (Issitt et al., 2019). Excess intracellular iron leads to mitochondrial dysfunction (Huang et al., 2009). This suggests that a positive feedback loop exists between iron overload and cell senescence that may aggravate brain homeostasis and contribute to cognitive dysfunction.

We observed a significant increase in the velocity, distance moved, and number of visits to borders, and a significant reduction in the percentage of time not moving in aged female mice, independently of the treatment, compared with aged male mice. Other studies found that aged females show enhanced anxiety‐like behavior than aged male mice (Connolly et al., 2021; McLean et al., 2022). It has been proposed that these sex differences in anxiety‐like behavior and reduced learning may be caused by enhanced expression of pro‐inflammatory molecules (TNFα, IL6) and neuroinflammation in the brain of aged female mice (Connolly et al., 2021; Mangold et al., 2017; Porcher et al., 2021). Overall, aged female mice show more locomotor activity than aged‐matched male mice (Connolly et al., 2021; Garvock‐de Montbrun et al., 2019; Haruyama et al., 2019; McLean et al., 2022), likely due to slightly reduced body weight in female compared with males. However, we did not find significant differences in body weight between sexes. Haruyama et al. proposed that the enhanced spontaneous locomotor activity in aged female mice can be caused by increased expression of an enzyme that prevent guanine oxidation in aged females, but not in males, independently of the body weight (Haruyama et al., 2019). Thus, there exist important sex differences in neuroinflammation and in the transcriptome between sexes that may affect anxiety‐like behavior and locomotor activity in aged mice.

Senescence in the brain vasculature can negatively affect BBB integrity (Yamazaki et al., 2016) and play a significant role in the pathogenesis of vascular dementia (Ueno et al., 2016). The increased levels of circulating markers of endothelial dysfunction are associated with age (50–75 y/o) and with cognitive dysfunction in humans (Heringa et al., 2014). In animal models, 10% of cerebromicrovascular endothelial cells become senescent in 28‐m/o mice (equivalent to 75 y/o in humans). Downregulation of *Sirt1*, which prevents senescence, in brain endothelial cells has been associated with enhanced permeability of BBB in the brains of aged mice and humans (Stamatovic et al., 2019). Soluble tau aggregates may also be responsible for inducing senescence in the brain vasculature and contribute to Alzheimer disease‐like vasculature deficits in mice (Hussong et al., 2023). This evidence supports that endothelial senescence is a major factor of BBB disruption and vascular dementia.

Our findings highlight sex‐dependent vulnerability to senescence by iron overload in the brain vasculature and in cultured primary CEC derived from aged mice. The major contributing factor to cell senescence is DNA damage (Durik et al., 2012; Yousefzadeh et al., 2021). From the existing literature, female sex is associated with reduced capacity for DNA repair in different organs and cell types (Broestl et al., 2022; Kfoury et al., 2018; Malorni et al., 2008; Rall‐Scharpf et al., 2021; Sun et al., 2014; Trzeciak et al., 2008), with one exception to this assumption found by Yousefzadeh et al. (2020). Walker et al. found that age (>55 y/o) has a greater effect on activating DNA damage response and senescence in aged women compared with aged men (Walker et al., 2016). We found that 150 μM FC significantly increased DNA damage in aged female mouse‐derived CEC, compared with female CEC treated either with 50 μM FC or a vehicle. However, cultured CEC derived from aged male mice showed enhanced DNA damage compared with female mouse‐derived CEC at basal conditions. Martin et al. discussed that we must be cautious with the general assumption that γH2AX represents DNA damage (Martin et al., 2014). Several studies found that γH2AX can occur in absence of DNA damage, and that may occur during replication (Ichijima et al., 2005; MacPhail et al., 2003; McManus & Hendzel, 2005; Tu et al., 2013), suggesting that cells with enhanced ability to proliferate may exhibit enhanced levels of γH2AX, with no correlation with DNA damage. Thus, measuring only DNA damage markers does not guarantee a positive correlation with senescence. From our data and considering also the reduced the expression of p16, p21, and IL6, male‐derived CEC are more resilient to FC‐induced senescence than female CEC.

Autophagy is an intracellular process that degrades and recycles cellular components as an adaptive strategy to environmental changes (Klionsky et al., 2021). Autophagy maintains the integrity of the BBB by regulating the levels of tight junction proteins, such as claudin‐5 (Yang et al., 2019). Either reduction (Hyun & Jung, 2014; Kaur et al., 2011; Rom et al., 2020) or accumulation (Feng et al., 2018; Gholami et al., 2022; Kakogiannos et al., 2020) of claudin‐5 results in the impairment of the BBB. Claudin‐5 localizes in the endothelial cell membrane to maintain the BBB permeability in conjunction with ZO‐1 (Jiao et al., 2011). However, it can be internalized in the cytosol by caveolin‐1‐mediated endocytosis; thus, being claudin‐5 excluded from brain endothelial borders, which causes BBB disruption (Liu et al., 2012). In the cytosol, claudin‐5 is degraded by autophagy. Indeed, autophagy eliminates aggregates of claudin‐5 that accumulate in the cytosol during hypoxia (Liu et al., 2016; Yang et al., 2021; Yu et al., 2021) or infections (Lin et al., 2022). However, autophagy impairment leads to accumulation of claudin‐5 in the cytosol of endothelial cells and impairs BBB (Liu et al., 2016; Yang et al., 2019). Importantly, iron overload negatively affects autophagy (Jahng et al., 2019; Uberti et al., 2020). We observed that autophagy was impaired by chronic administration of FC in aged female mice. Similarly, claudin‐5 levels were enhanced in the brains of aged female mice treated with FC, but not in male mice. Thus, autophagy impairment by FC may lead to claudin‐5 accumulation, and this could lead to BBB breakdown.

ROBO4 is a transmembrane receptor protein that is specifically expressed in endothelial cells (Huminiecki et al., 2002). ROBO4 is mostly known as a regulator of endothelial cell migration and proliferation (Dai et al., 2019). Dysregulated expression of *ROBO4*, either upregulation or downregulation, has been importantly associated with angiogenesis sites and in cancer tissues (Huminiecki et al., 2002; Yamanaka et al., 2022; Yeo et al., 2022). In preclinical studies, *Robo4* downregulation enhances permeability in cultured endothelial cells (Bekes et al., 2017; Cai et al., 2015). Robo4 has also been involved in pro‐inflammatory responses that ultimately lead to cell senescence. The pro‐inflammatory cytokine TNF‐α can promote the activation of NF‐κB, which binds to the promoter of *Robo4*, and thus enhances the expression of *Robo4*, likely as a compensatory mechanism to mitigate deleterious effects of TNFα (Tanaka et al., 2017). Robo4 interacts with TNFreceptor‐associated factor 7 and this complex can prevent hyperpermeability induced by TNF‐α by inhibiting cytoplasmic internalization of the adherens junction protein VE‐cadherin (Shirakura et al., 2019). We found that IL6 is upregulated in female mouse‐derived CEC and in *Robo4* downregulated male‐derived CEC treated with 150 μM FC, which supports the anti‐inflammatory role of Robo4. However, a study found that *Robo4* downregulation prevents LPS‐induced IL6 production by inhibiting the synthesis of granulocyte macrophage colony‐stimulating factor (Shirakura et al., 2018). Thus, Robo4 may have a dual role in inflammatory responses likely depending on cellular environments.

Our study provides evidence that sex differences exist in the brain function and, particularly, in the brain vasculature of aged mice with chronic administration of FC, and that *Robo4* downregulation is a senescence‐inducing factor in combination with FC. This study highlights the necessity to evaluate the risk of brain vascular impairment and dementia in female patients and in patients with ROBO4 variants. Importantly, developing therapeutical approaches to prevent endothelial senescence is a strategy to protect brain vasculature and mitigate vascular dementia in the elderly population.

## AUTHOR CONTRIBUTIONS

Brian Noh isolated and cultured CEC and performed the majority of experiments and gathered data. Maria Pilar Blasco‐Conesa, Syed Mushfiqur Rahman, and Sheelu Monga performed individual experiments and analyzed data. Gary Guzman and Rodney Ritzel performed flow cytometry experiments. Yun‐Ju Lai processed CEC samples for RNA‐seq. Bhanu Priya Ganesh, Akihiko Urayama, and Louise D. McCullough advised on the study. Jose Felix Moruno‐Manchon designed the study, prepared figures, and wrote the manuscript.

## FUNDING INFORMATION

This research was conducted with the financial support to the J.F.M.M.'s lab from the NIA (R21AG075750, J.F.M.M.), the Texas Alzheimer's Research and Care Consortium (#957578, J.F.M.M.), the American Heart Association (#856061, J.F.M.M.), and start‐up funds from the University of Texas Health Science Center at Houston McGovern Medical School (J.F.M.M).

## CONFLICT OF INTEREST STATEMENT

All authors declare no competing financial interests.

## Supporting information


FigureS1

FigureS2

FigureS3

FigureS4

FigureS5

FigureS6

FigureS7

FigureS8

TableS1

## Data Availability

The data that support the findings of this study are openly available in Harvard Dataverse at https://doi.org/10.7910/DVN/B9YKGC.
