# Supplementary figures and images for: Iron overload induces cerebral endothelial senescence in aged mice and in primary culture in a sex‐dependent manner
Source: Aging Cell. 2023 Sep 7;22(11):e13977. doi: 10.1111/acel.13977 (PMC10652299; doi:10.1111/acel.13977)

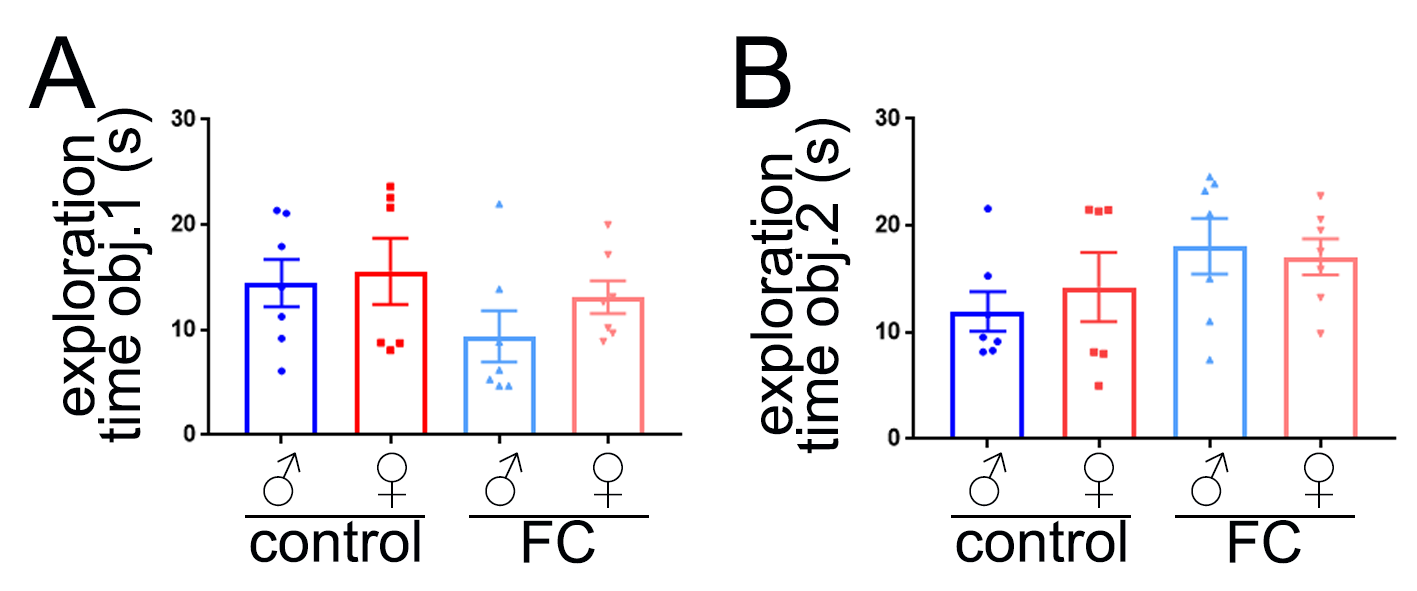

Supplement: Supplementary file 1 — FigureS1 [file ACEL-22-e13977-s008.png]

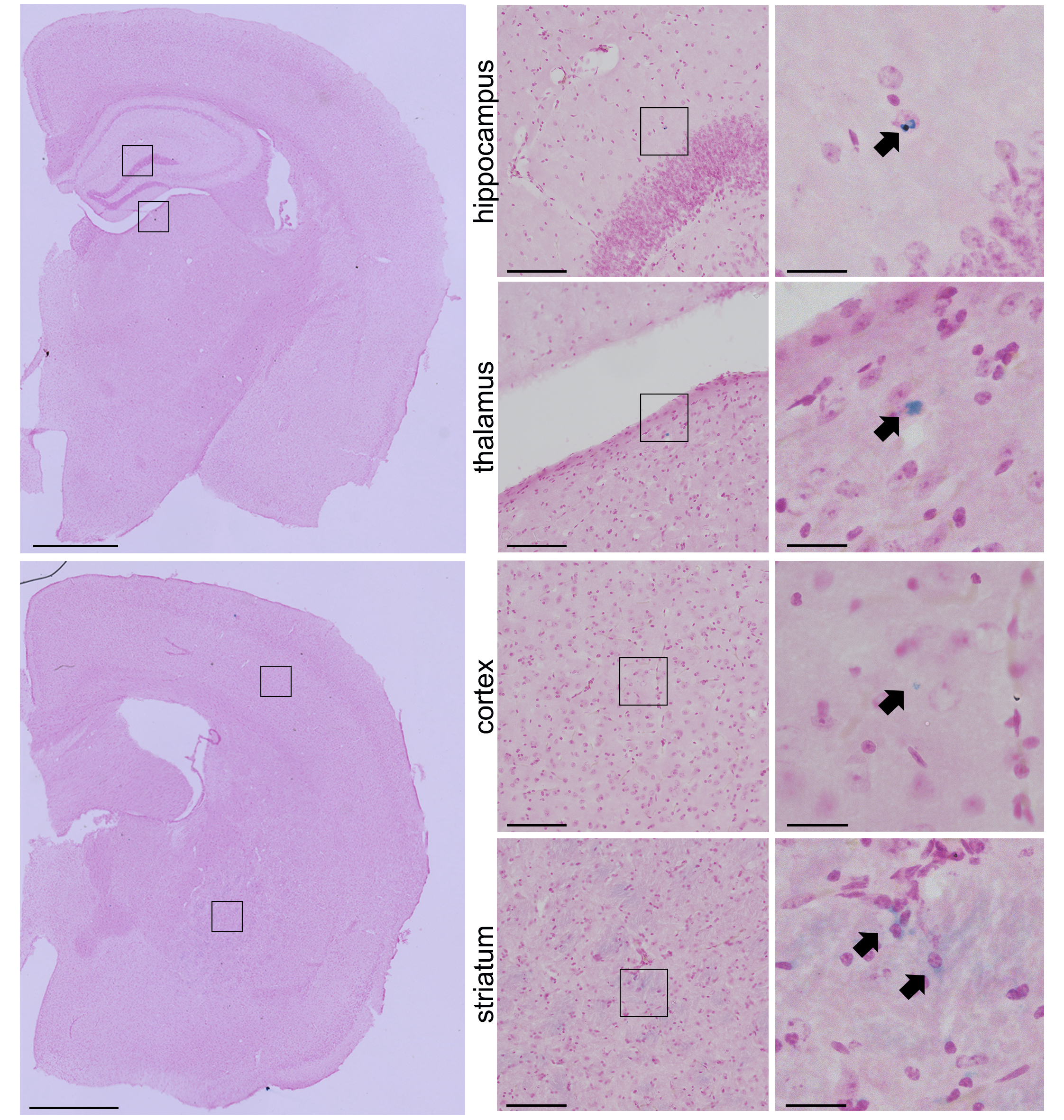

Supplement: Supplementary file 2 — FigureS2 [file ACEL-22-e13977-s004.png]

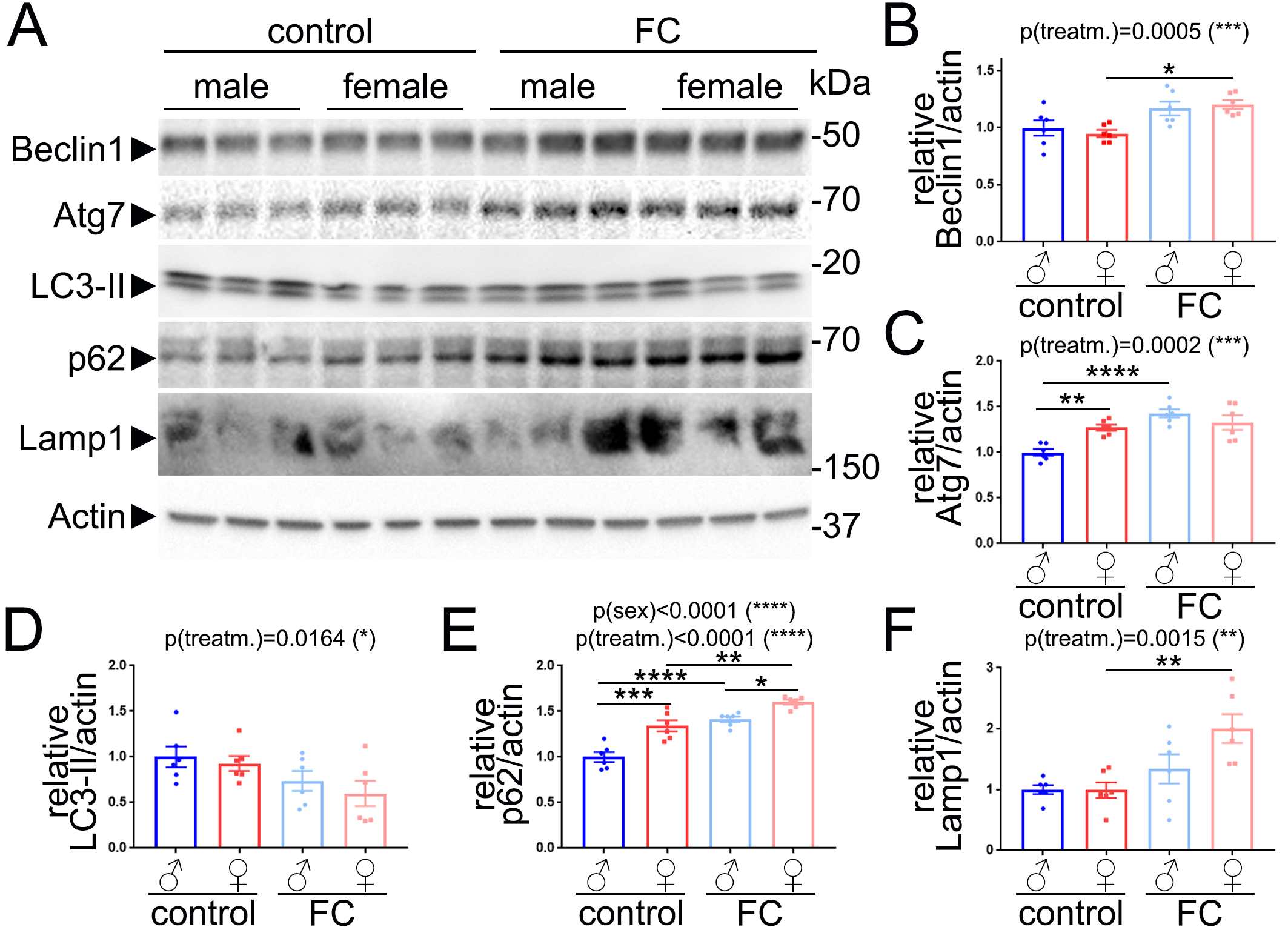

Supplement: Supplementary file 3 — FigureS3 [file ACEL-22-e13977-s005.png]

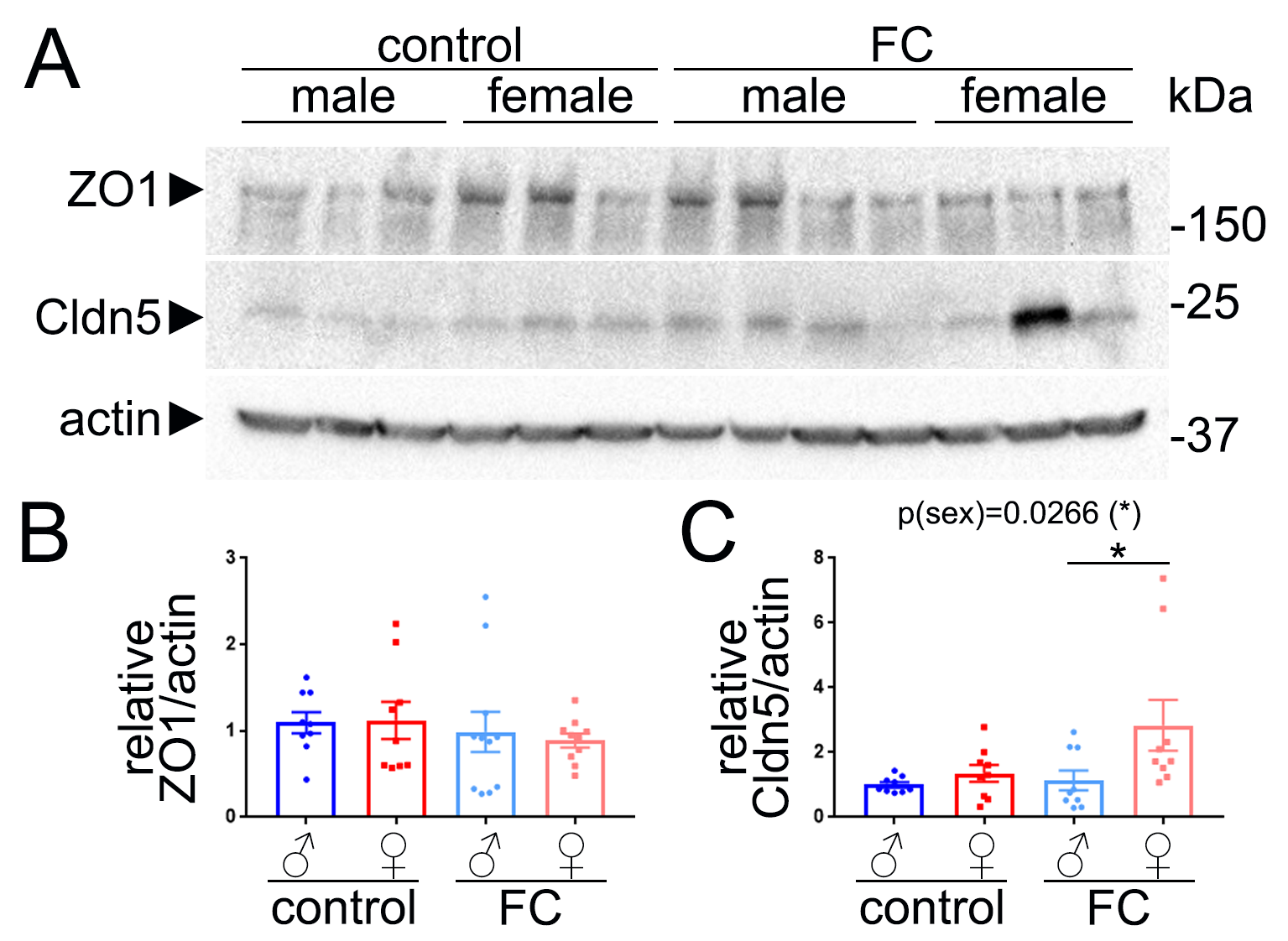

Supplement: Supplementary file 4 — FigureS4 [file ACEL-22-e13977-s003.png]

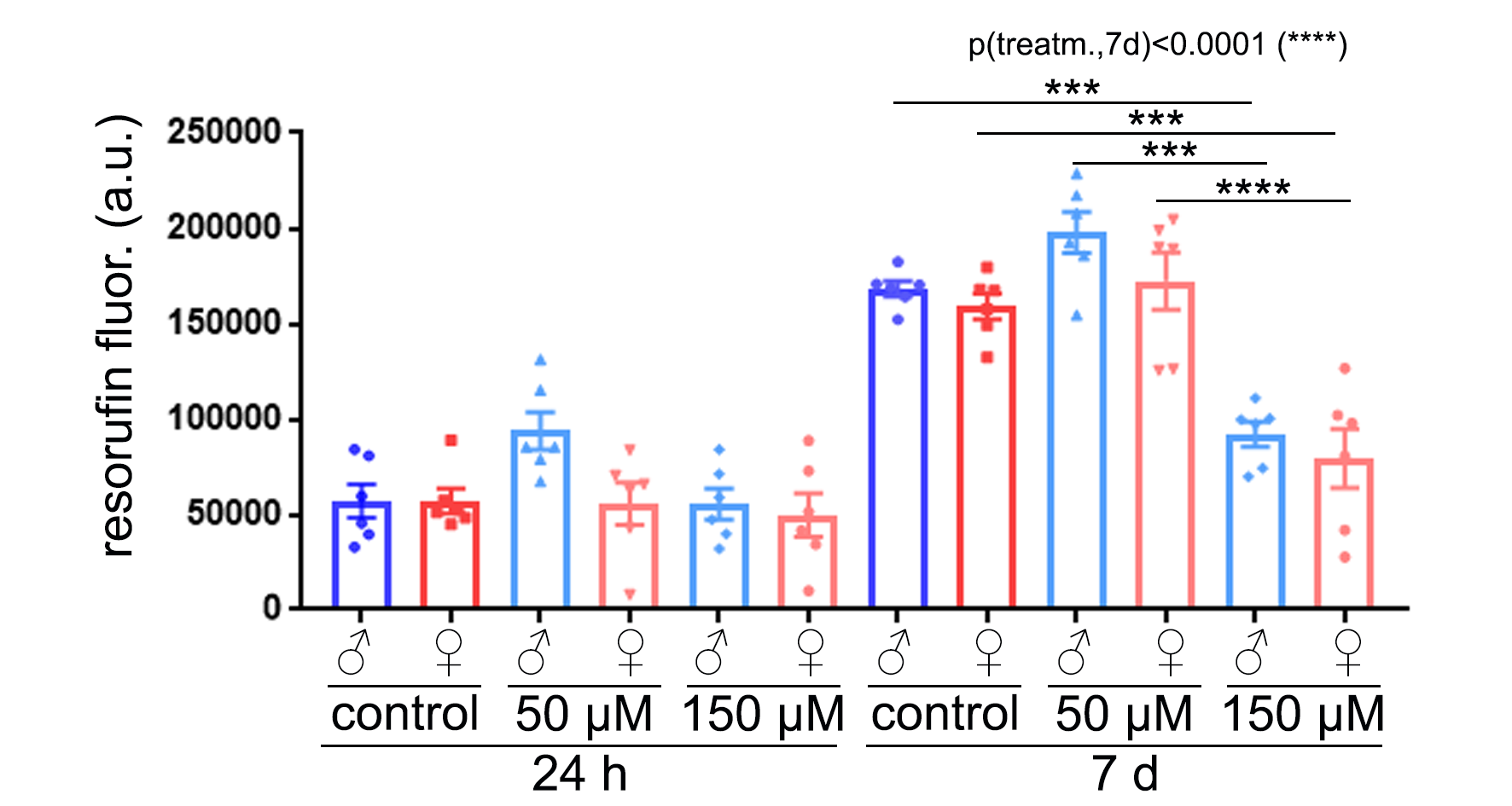

Supplement: Supplementary file 5 — FigureS5 [file ACEL-22-e13977-s006.png]

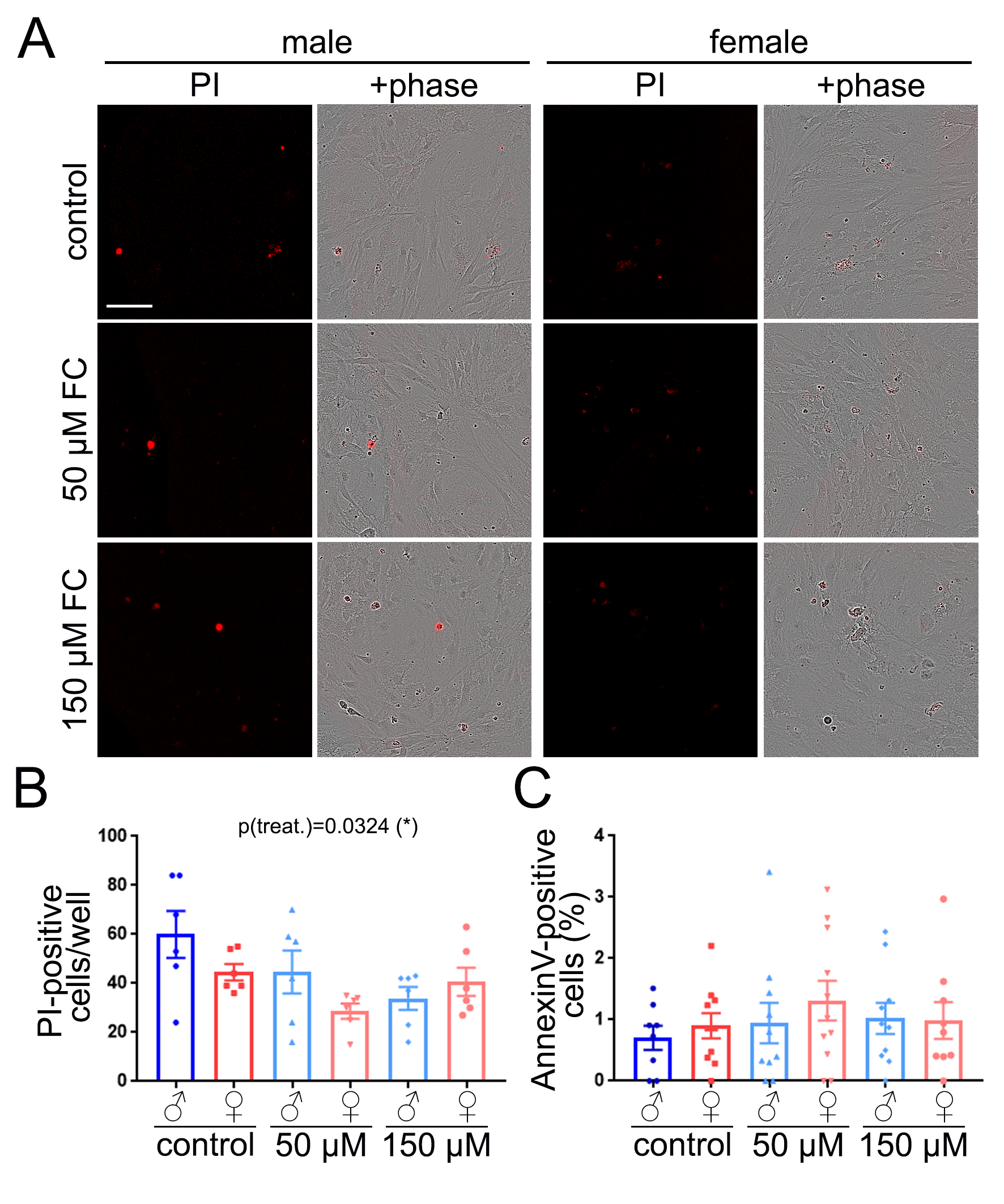

Supplement: Supplementary file 6 — FigureS6 [file ACEL-22-e13977-s007.png]

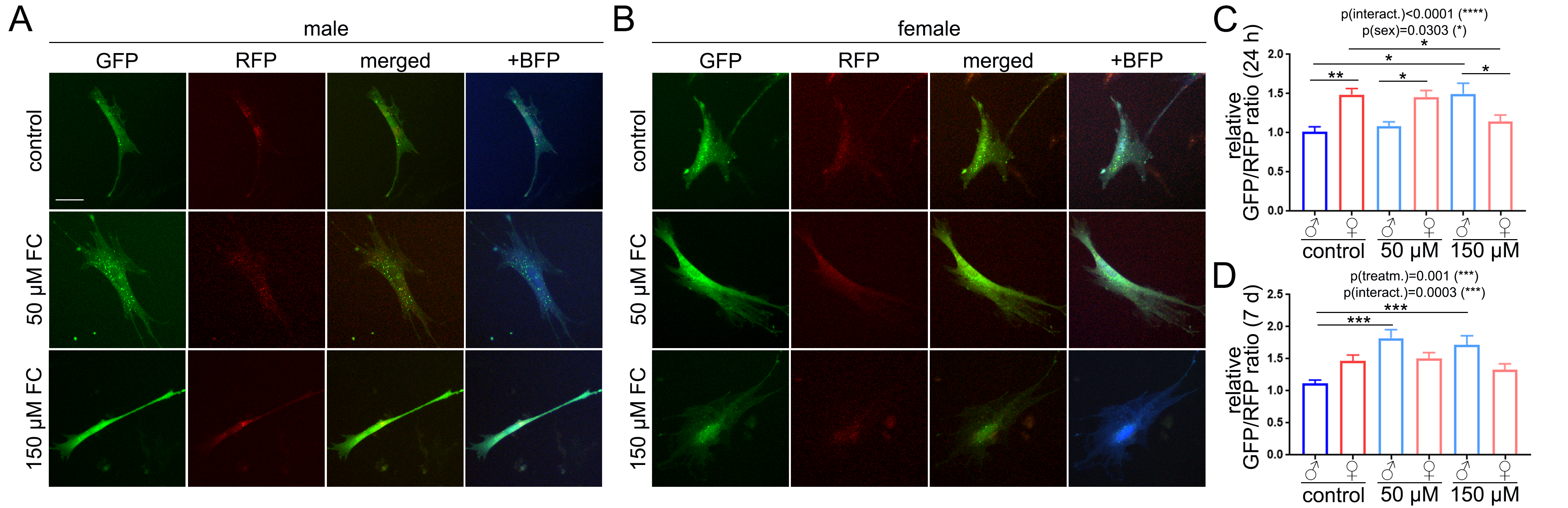

Supplement: Supplementary file 7 — FigureS7 [file ACEL-22-e13977-s001.png]

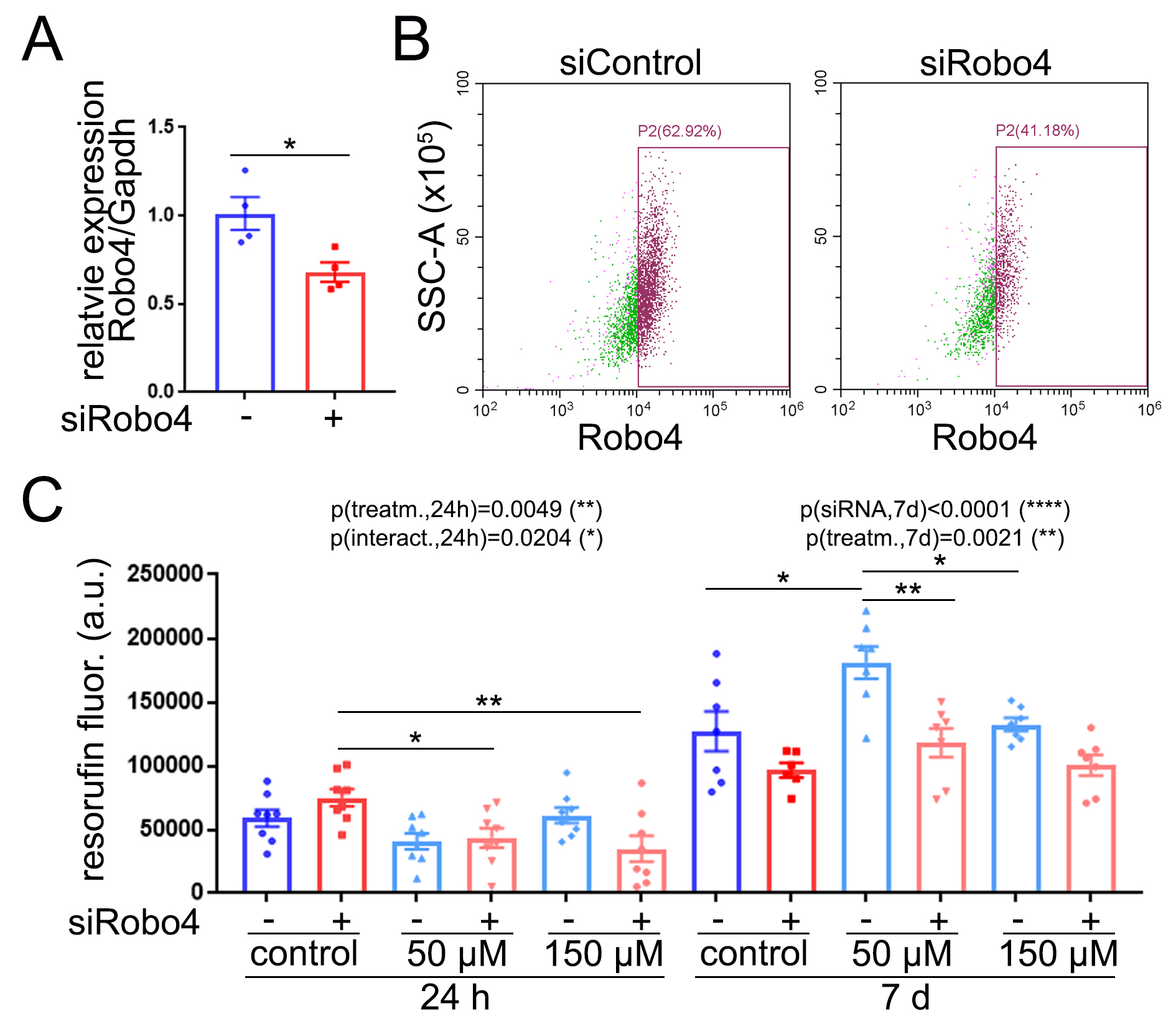

Supplement: Supplementary file 8 — FigureS8 [file ACEL-22-e13977-s009.png]
