# Supplementary material for: Iron overload induces cerebral endothelial senescence in aged mice and in primary culture in a sex‐dependent manner
Source: Aging Cell. 2023 Sep 7;22(11):e13977. doi: 10.1111/acel.13977 (PMC10652299; doi:10.1111/acel.13977)
Supplement: Supplementary file 9 — TableS1 [file ACEL-22-e13977-s002.docx]

**Supplementary Table 1. Tabular results of 2-way ANOVA.**

| Tabular results of 2-way ANOVA for the Figure 1 | | |
| --- | --- | --- |
| **Body weight (Fig 1B)** | | |
| Source of variation | F (DFn, DFd) | P value (summary) |
| Interaction | F (1, 30) = 0.07204 | P=0.7902 (n.s.) |
| Sex | F (1, 30) = 0.2427 | P=0.6259 (n.s.) |
| Treatment | F (1, 30) = 0.3616 | P=0.5521 (n.s.) |
| **Velocity (Fig 1C)** | | |
| Source of variation | F (DFn, DFd) | P value (summary) |
| Interaction | F (1, 24) = 0.5946 | P=0.4482 (n.s.) |
| Sex | F (1, 24) = 8.18 | P=0.0086 (**) |
| Treatment | F (1, 24) = 0.1418 | P=0.7098 (n.s.) |
| **Distance moved (Fig 1D)** | | |
| Source of variation | F (DFn, DFd) | P value (summary) |
| Interaction | F (1, 24) = 0.4691 | P=0.5000 (n.s.) |
| Sex | F (1, 24) = 4.277 | P=0.0496 (*) |
| Treatment | F (1, 24) = 0.05906 | P=0.8100 (n.s.) |
| **% Time not moving (Fig 1E)** | | |
| Source of variation | F (DFn, DFd) | P value (summary) |
| Interaction | F (1, 24) = 0.1049 | P=0.7488 (n.s.) |
| Sex | F (1, 24) = 3.96 | P=0.0581 (n.s.) |
| Treatment | F (1, 24) = 0.1896 | P=0.6672 (n.s.) |
| **% Time in the center (Fig 1F)** | | |
| Source of variation | F (DFn, DFd) | P value (summary) |
| Interaction | F (1, 24) = 0.3701 | P=0.5487 (n.s.) |
| Sex | F (1, 24) = 0.2866 | P=0.5973 (n.s.) |
| Treatment | F (1, 24) = 1.187 | P=0.2867 (n.s.) |
| **Visits to center (Fig 1G)** | | |
| Source of variation | F (DFn, DFd) | P value (summary) |
| Interaction | F (1, 24) = 0.1802 | P=0.6750 (n.s.) |
| Sex | F (1, 24) = 0.7773 | P=0.3867 (n.s.) |
| Treatment | F (1, 24) = 0.03199 | P=0.8596 (n.s.) |
| **% Time in borders (Fig 1H)** | | |
| Source of variation | F (DFn, DFd) | P value (summary) |
| Interaction | F (1, 24) = 0.44 | P=0.5134 (n.s.) |
| Sex | F (1, 24) = 0.4148 | P=0.5256 (n.s.) |
| Treatment | F (1, 24) = 1.176 | P=0.2889 (n.s.) |
| **Visits to borders (Fig 1I)** | | |
| Source of variation | F (DFn, DFd) | P value (summary) |
| Interaction | F (1, 24) = 0.03972 | P=0.8437 (n.s.) |
| Sex | F (1, 24) = 4.73 | P=0.0397 (*) |
| Treatment | F (1, 24) = 0.232 | P=0.6344 (n.s.) |
| **% Alternation (Fig 1J)** | | |
| Source of variation | F (DFn, DFd) | P value (summary) |
| Interaction | F (1, 19) = 0.06929 | P=0.7952 (n.s.) |
| Sex | F (1, 19) = 0.481 | P=0.4964 (n.s.) |
| Treatment | F (1, 19) = 0.4567 | P=0.5073 (n.s.) |
| **Recognition index (Fig 1K)** | | |
| Source of variation | F (DFn, DFd) | P value (summary) |
| Interaction | F (1, 23) = 5.231 | P=0.0317 (*) |
| Sex | F (1, 23) = 2.774 | P=0.1094 (n.s.) |
| Treatment | F (1, 23) = 0.1955 | P=0.6625 (n.s.) |
| **% Inactive time (Fig 1L)** | | |
| **Source of variation** | **F (DFn, DFd)** | **P value (summary)** |
| Interaction | F (1, 20) = 1.389 | P=0.2525 (n.s.) |
| Sex | F (1, 20) = 0.8495 | P=0.3677 (n.s.) |
| Treatment | F (1, 20) = 15.87 | P=0.0007 (***) |
| Tabular results of 2-way ANOVA for the Figure 2 | | |
| **Number of Prussian blue-positive deposits (Fig 2B)** | | |
| **Source of variation** | **F (DFn, DFd)** | **P value (summary)** |
| Interaction | F (1, 12) = 2.286 | P=0.1564 (n.s.) |
| Sex | F (1, 12) = 2.182 | P=0.1654 (n.s.) |
| Treatment | F (1, 12) = 9.396 | P=0.0098 (**) |
| **Iron content in blood cells (Fig 2B)** | | |
| Source of variation | F (DFn, DFd) | P value (summary) |
| Interaction | F (1, 15) = 0.03347 | P=0.8573 (n.s.) |
| Sex | F (1, 15) = 0.0132 | P=0.9101 (n.s.) |
| Treatment | F (1, 15) = 0.06625 | P=0.8004 (n.s.) |
| **Iron content in brains (Fig 2C)** | | |
| Source of variation | F (DFn, DFd) | P value (summary) |
| Interaction | F (1, 24) = 0.5288 | P=0.4741 (n.s.) |
| Sex | F (1, 24) = 1.994 | P=0.1708 (n.s.) |
| Treatment | F (1, 24) = 62.24 | P<0.0001 (***) |
| Tabular results of 2-way ANOVA for the Figure 3 | | |
| **% γH2AX-positive area (Fig 3B)** | | |
| Source of variation | F (DFn, DFd) | P value (summary) |
| Interaction | F (1, 16) = 0.1049 | P=0.7503 (n.s.) |
| Sex | F (1, 16) = 14.31 | P=0.0016 (**) |
| Treatment | F (1, 16) = 2.479 | P=0.1349 (n.s.) |
| **γH2AX-puncta index (Fig 3E)** | | |
| Source of variation | F (DFn, DFd) | P value (summary) |
| Interaction | F (1, 1671) = 2.129 | P=0.1448 (n.s.) |
| Sex | F (1, 1671) = 1.89 | P=0.1694 (n.s.) |
| Treatment | F (1, 1671) = 7.715 | P=0.0055 (**) |
| **p16/Gapdh (Fig 3F)** | | |
| **Source of variation** | **F (DFn, DFd)** | **P value (summary)** |
| Interaction | F (1, 12) = 0.004062 | P=0.9502 (n.s.) |
| Sex | F (1, 12) = 0.07554 | P=0.7881 (n.s.) |
| Treatment | F (1, 12) = 0.1973 | P=0.6648 (n.s.) |
| **p21/Gapdh (Fig 3G)** | | |
| **Source of variation** | **F (DFn, DFd)** | **P value (summary)** |
| Interaction | F (1, 12) = 5.625 | P=0.0353 (*) |
| Sex | F (1, 12) = 13.37 | P=0.0033 (**) |
| Treatment | F (1, 12) = 2.419 | P=0.1458 (n.s.) |
| **IL-6/Gapdh (Fig 3H)** | | |
| **Source of variation** | **F (DFn, DFd)** | **P value (summary)** |
| Interaction | F (1, 12) = 0.005916 | P=0.9400 (n.s.) |
| Sex | F (1, 12) = 0.4268 | P=0.5259 (n.s.) |
| Treatment | F (1, 12) = 11.85 | P=0.0049 (**) |
| Tabular results of 2-way ANOVA for the Figure 5 | | |
| **Wound closure (Fig 5D)** | | |
| **Source of variation** | **F (DFn, DFd)** | **P value (summary)** |
| Interaction | F (2, 30) = 12.96 | P<0.0001 (****) |
| Sex | F (1, 30) = 4.448 | P=0.0434 (*) |
| Treatment | F (2, 30) = 26.03 | P<0.0001 (****) |
| **γH2AX puncta index (Fig 5F)** | | |
| **Source of variation** | **F (DFn, DFd)** | **P value (summary)** |
| Interaction | F (2, 14163) = 51 | P<0.0001 (****) |
| Sex | F (1, 14163) = 85.35 | P<0.0001 (****) |
| Treatment | F (2, 14163) = 61.93 | P<0.0001 (****) |
| **p16/Gapdh (Fig 5G)** | | |
| **Source of variation** | **F (DFn, DFd)** | **P value (summary)** |
| Interaction | F (2, 18) = 2.352 | P=0.1237 (n.s.) |
| Sex | F (1, 18) = 44.55 | P<0.0001 (****) |
| Treatment | F (2, 18) = 2.852 | P=0.0839 (n.s.) |
| **p21/Gapdh (Fig 5H)** | | |
| **Source of variation** | **F (DFn, DFd)** | **P value (summary)** |
| Interaction | F (2, 18) = 4.988 | P=0.0189 (*) |
| Sex | F (1, 18) = 31.71 | P<0.0001 (****) |
| Treatment | F (2, 18) = 7.805 | P=0.0036 (**) |
| **IL-6/Gapdh (Fig 5I)** | | |
| **Source of variation** | **F (DFn, DFd)** | **P value (summary)** |
| Interaction | F (2, 18) = 24.13 | P<0.0001 (****) |
| Sex | F (1, 18) = 153.4 | P<0.0001 (****) |
| Treatment | F (2, 18) = 25.92 | P<0.0001 (****) |
| Tabular results of 2-way ANOVA for the Figure 6 | | |
| **Wound closure (Fig 6E)** | | |
| **Source of variation** | **F (DFn, DFd)** | **P value (summary)** |
| Interaction | F (2, 23) = 11.52 | P=0.0003 (***) |
| siRNA | F (1, 23) = 19.38 | P=0.0002 (***) |
| Treatment | F (2, 23) = 17.97 | P<0.0001 (****) |
| **γH2AX puncta index (Fig 6F)** | | |
| **Source of variation** | **F (DFn, DFd)** | **P value (summary)** |
| Interaction | F (2, 3413) = 7.063 | P=0.0009 (***) |
| siRNA | F (1, 3413) = 0.06468 | P=0.7993 (n.s.) |
| Treatment | F (2, 3413) = 0.6836 | P=0.5049 (n.s.) |
| **p16/Gapdh (Fig 6G)** | | |
| **Source of variation** | **F (DFn, DFd)** | **P value (summary)** |
| Interaction | F (2, 26) = 2.133 | P=0.1387 (n.s.) |
| siRNA | F (1, 26) = 4.917 | P=0.0355 (*) |
| Treatment | F (2, 26) = 2.997 | P=0.0674 (n.s.) |
| **p21/Gapdh (Fig 6H)** | | |
| **Source of variation** | **F (DFn, DFd)** | **P value (summary)** |
| Interaction | F (2, 25) = 2.118 | P=0.1413 (n.s.) |
| siRNA | F (1, 25) = 4.172 | P=0.0518 (n.s.) |
| Treatment | F (2, 25) = 2.749 | P=0.0834 (n.s.) |
| **IL-6/Gapdh (Fig 6I)** | | |
| **Source of variation** | **F (DFn, DFd)** | **P value (summary)** |
| Interaction | F (2, 25) = 2.867 | P=0.0757 (n.s.) |
| siRNA | F (1, 25) = 7.153 | P=0.0130 (*) |
| Treatment | F (2, 25) = 4.223 | P=0.0263 (*) |
| Tabular results of 2-way ANOVA for the Supplementary Figure 1 | | |
| **Exploration time in object 1 (Sup Fig 1A)** | | |
| **Source of variation** | **F (DFn, DFd)** | **P value (summary)** |
| Interaction | F (1, 23) = 0.2998 | P=0.5893 (n.s.) |
| Sex | F (1, 23) = 1.06 | P=0.3138 (n.s.) |
| Treatment | F (1, 23) = 2.536 | P=0.1250 (n.s.) |
| **Exploration time in object 2 (Sup Fig 1B)** | | |
| **Source of variation** | **F (DFn, DFd)** | **P value (summary)** |
| Interaction | F (1, 23) = 0.4741 | P=0.4980 (n.s.) |
| Sex | F (1, 23) = 0.07538 | P=0.7861 (n.s.) |
| Treatment | F (1, 23) = 3.565 | P=0.0717 (n.s.) |
| Tabular results of 2-way ANOVA for the Supplementary Figure 3 | | |
| **Beclin1/actin (Sup Fig 3B)** | | |
| **Source of variation** | **F (DFn, DFd)** | **P value (summary)** |
| Interaction | F (1, 20) = 0.671 | P=0.4224 (n.s.) |
| Sex | F (1, 20) = 0.01976 | P=0.8896 (n.s.) |
| Treatment | F (1, 20) = 17.09 | P=0.0005 (***) |
| **Atg7/actin (Sup Fig 3C)** | | |
| **Source of variation** | **F (DFn, DFd)** | **P value (summary)** |
| Interaction | F (1, 20) = 12.47 | P=0.0021 (**) |
| Sex | F (1, 20) = 2.695 | P=0.1163 (n.s.) |
| Treatment | F (1, 20) = 21.69 | P=0.0002 (***) |
| **LC3-II/actin (Sup Fig 3D)** | | |
| **Source of variation** | **F (DFn, DFd)** | **P value (summary)** |
| Interaction | F (1, 20) = 0.08175 | P=0.7779 (n.s.) |
| Sex | F (1, 20) = 0.8508 | P=0.3673 (n.s.) |
| Treatment | F (1, 20) = 6.861 | P=0.0164 (*) |
| **p62/actin (Sup Fig 3E)** | | |
| **Source of variation** | **F (DFn, DFd)** | **P value (summary)** |
| Interaction | F (1, 20) = 2.813 | P=0.1091 (n.s.) |
| Sex | F (1, 20) = 34.46 | P<0.0001 (****) |
| Treatment | F (1, 20) = 54.98 | P<0.0001 (****) |
| **Lamp1/actin (Sup Fig3F)** | | |
| **Source of variation** | **F (DFn, DFd)** | **P value (summary)** |
| Interaction | F (1, 20) = 3.313 | P=0.0838 (n.s.) |
| Sex | F (1, 20) = 3.148 | P=0.0912 (n.s.) |
| Treatment | F (1, 20) = 13.5 | P=0.0015 (**) |
| Tabular results of 2-way ANOVA for the Supplementary Figure 4 | | |
| **ZO1/actin (Sup Fig 4B)** | | |
| **Source of variation** | **F (DFn, DFd)** | **P value (summary)** |
| Interaction | F (1, 35) = 0.1277 | P=0.7230 (n.s.) |
| Sex | F (1, 35) = 0.04257 | P=0.8377 (n.s.) |
| Treatment | F (1, 35) = 0.9185 | P=0.3444 (n.s.) |
| **Claudin-5/actin (Sup Fig 4C)** | | |
| **Source of variation** | **F (DFn, DFd)** | **P value (summary)** |
| Interaction | F (1, 32) = 2.329 | P=0.1368 (n.s.) |
| Sex | F (1, 32) = 5.407 | P=0.0266 (*) |
| Treatment | F (1, 32) = 3.364 | P=0.0760 (n.s.) |
| Tabular results of 2-way ANOVA for the Supplementary Figure 5 | | |
| **CellTiter fluorescence 24h (Sup Fig 5)** | | |
| **Source of variation** | **F (DFn, DFd)** | **P value (summary)** |
| Interaction | F (2, 30) = 2.39 | P=0.1089 (n.s.) |
| Sex | F (1, 30) = 3.604 | P=0.0673 (n.s.) |
| Treatment | F (2, 30) = 3.045 | P=0.0625 (n.s.) |
| **CellTiter fluorescence 7d (Sup Fig 5)** | | |
| **Source of variation** | **F (DFn, DFd)** | **P value (summary)** |
| Interaction | F (2, 30) = 0.3179 | P=0.7301 (n.s.) |
| Sex | F (1, 30) = 3.267 | P=0.0807 (n.s.) |
| Treatment | F (2, 30) = 47.85 | P<0.0001 (****) |
| Tabular results of 2-way ANOVA for the Supplementary Figure 6 | | |
| **Propidium iodide-positive cells/well (Sup Fig 6B)** | | |
| **Source of variation** | **F (DFn, DFd)** | **P value (summary)** |
| Interaction | F (2, 30) = 2.101 | P=0.1399 (n.s.) |
| Sex | F (1, 30) = 2.503 | P=0.1241 (n.s.) |
| Treatment | F (2, 30) = 3.853 | P=0.0324 (n.s.) |
| **AnnexinV-positive cells (%) (Sup Fig 5C)** | | |
| **Source of variation** | **F (DFn, DFd)** | **P value (summary)** |
| Interaction | F (2, 52) = 0.2542 | P=0.7765 (n.s.) |
| Sex | F (1, 52) = 0.5712 | P=0.4532 (n.s.) |
| Treatment | F (2, 52) = 0.6696 | P=0.5163 (n.s.) |
| Tabular results of 2-way ANOVA for the Supplementary Figure 7 | | |
| **Autophagy flux 24h (Sup Fig 7C)** | | |
| **Source of variation** | **F (DFn, DFd)** | **P value (summary)** |
| Interaction | F (2, 513) = 11.7 | P<0.0001 (****) |
| Sex | F (1, 513) = 4.72 | P=0.0303 (*) |
| Treatment | F (2, 513) = 0.3079 | P=0.7351 (n.s.) |
| **Autophagy flux 7d (Sup Fig 7D)** | | |
| **Source of variation** | **F (DFn, DFd)** | **P value (summary)** |
| Interaction | F (2, 502) = 8.202 | P=0.0003 (***) |
| Sex | F (1, 502) = 2.133 | P=0.1447 (n.s.) |
| Treatment | F (2, 502) = 7.021 | P=0.0010 (***) |
| Tabular results of 2-way ANOVA for the Supplementary Figure 8 | | |
| **CellTiter fluorescence 24h (Sup Fig 7C)** | | |
| **Source of variation** | **F (DFn, DFd)** | **P value (summary)** |
| Interaction | F (2, 42) = 4.275 | P=0.0204 (*) |
| siRNA | F (1, 42) = 0.1712 | P=0.6811 (n.s.) |
| Treatment | F (2, 42) = 6.055 | P=0.0049 (**) |
| **CellTiter fluorescence 7d (Sup Fig 7C)** | | |
| **Source of variation** | **F (DFn, DFd)** | **P value (summary)** |
| Interaction | F (2, 35) = 1.464 | P=0.2451 (n.s.) |
| siRNA | F (1, 35) = 22.85 | P<0.0001 (****) |
| Treatment | F (2, 35) = 7.371 | P=0.0021 (**) |
